# Energy conversion and storage via photoinduced polarization change in non-ferroelectric molecular [CoGa] crystals

**DOI:** 10.1038/s41467-023-39127-8

**Published:** 2023-06-09

**Authors:** Pritam Sadhukhan, Shu-Qi Wu, Shinji Kanegawa, Sheng-Qun Su, Xiaopeng Zhang, Takumi Nakanishi, Jeremy Ian Long, Kaige Gao, Rintaro Shimada, Hajime Okajima, Akira Sakamoto, Joy G. Chiappella, Myron S. Huzan, Thomas Kroll, Dimosthenis Sokaras, Michael L. Baker, Osamu Sato

**Affiliations:** 1 Institute for Materials Chemistry and Engineering & IRCCS, Kyushu University, 744 Motooka, Nishi-ku, Fukuoka, 819-0395 Japan; 2 College of Physical Science and Technology, Yangzhou University, Jiangsu, 225009 P. R. China; 3 Graduate School of Science and Engineering, Aoyama Gakuin University, 5-10-1 Fuchinobe, Chuo-ku, Sagamihara, Kanagawa 252-5258 Japan; 4 The Department of Chemistry, The University of Manchester, Manchester, M13 9PL UK; 5 The Department of Chemistry, The University of Manchester at Harwell, Didcot, OX11 0FA UK; 6 Stanford Synchrotron Radiation Lightsource, SLAC National Accelerator Laboratory, Stanford University, Menlo Park, 94025 CA USA

**Keywords:** Electronic materials, Optical materials, Electronic devices

## Abstract

To alleviate the energy and environmental crisis, in the last decades, energy harvesting by utilizing optical control has emerged as a promising solution. Here we report a polar crystal that exhibits photoenergy conversion and energy storage upon light irradiation. The polar crystal consists of dinuclear [CoGa] molecules, which are oriented in a uniform direction inside the crystal lattice. Irradiation with green light induces a directional intramolecular electron transfer from the ligand to a low-spin Co^III^ centre, and the resultant light-induced high-spin Co^II^ excited state is trapped at low temperature, realizing energy storage. Additionally, electric current release is observed during relaxation from the trapped light-induced metastable state to the ground state, because the intramolecular electron transfer in the relaxation process is accompanied with macroscopic polarization switching at the single-crystal level. It demonstrates that energy storage and conversion to electrical energy is realized in the [CoGa] crystals, which is different from typical polar pyroelectric compounds that exhibit the conversion of thermal energy into electricity.

## Abstract (web summary)

Energy harvesting by utilizing optical control has emerged as a promising solution to alleviate energy and environmental crisis. However, it is challenging to realise nano-scale energy storage and conversion in the same material. Here the authors report a nonferroelectric molecular [CoGa] crystal that uses light as an external stimulus to exhibit photoenergy conversion and energy storage properties.

## Introduction

**Fig. 1:**
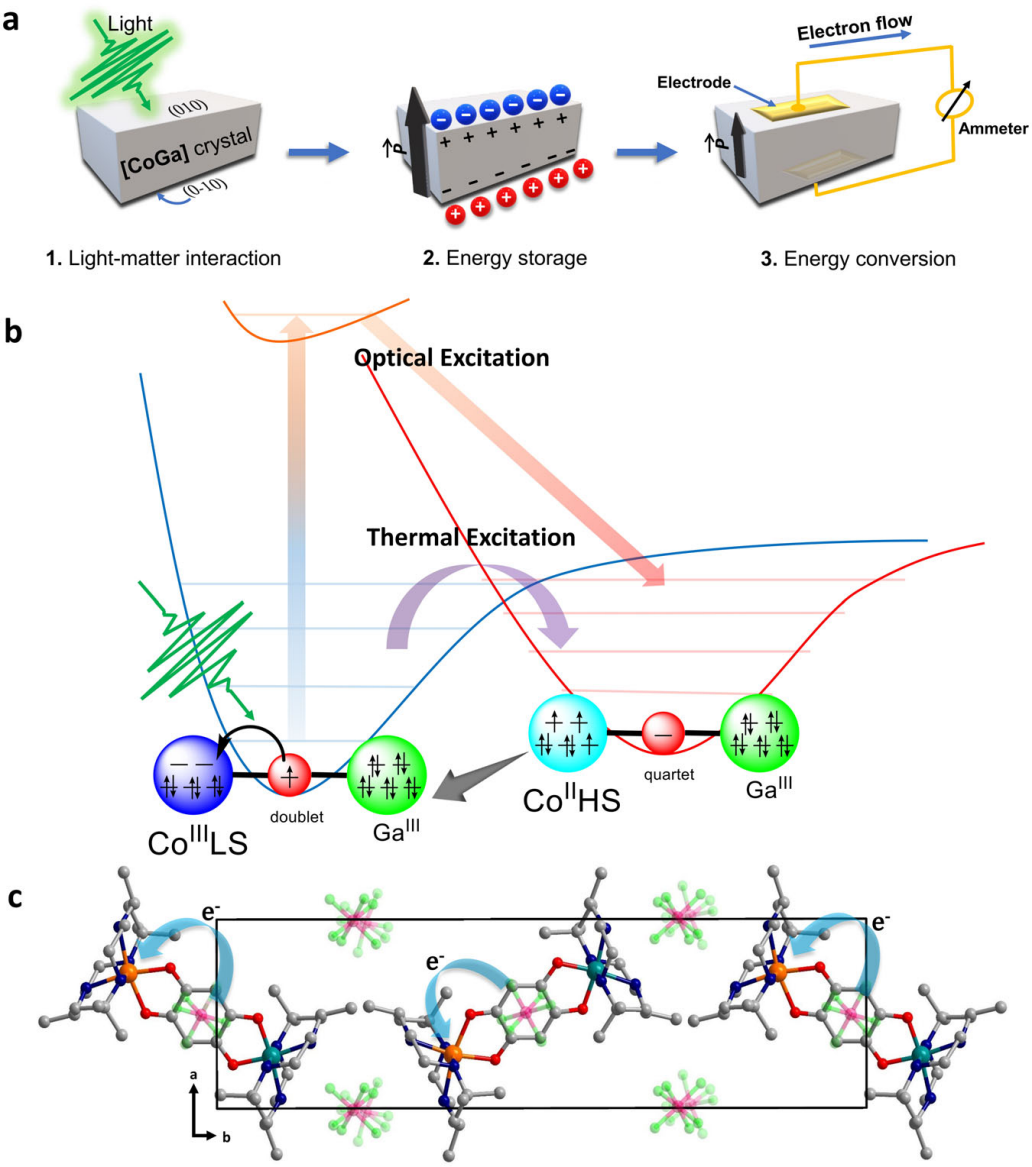
Effect of light on [CoGa] crystals at the microscopic and macroscopic scale. **a** Schematic representation of the energy storage and conversion mechanism upon light irradiation. Due to the crystallization in a polar space group, molecular dipole switching is manifested as macroscopic polarization switching upon light excitation which produces an excited polarization state (higher polarization state) with long lifetime (energy storage). Relaxation from the trapped metastable state induces macroscopic polarization switching which generates an electric current in the external circuit (energy conversion). **b** Mechanism of the light and thermally induced polarization change; electron transfer between cobalt and ligand. **c** Crystal packing of the complex which favors unidirectional electron transfer inside the lattice. (color code: cobalt—orange, gallium—dark green, carbon—grey, oxygen—red, nitrogen—blue, phosphorus—pink, fluorine—light green, hydrogen atoms are omitted for structural clarity).

Energy conversion is a prime concern of the scientific community and industrial sectors around the world^1–3^. Among the various stimuli, light is a clean energy source which is both safe and abundant, and it also allows for a precise remote control since the light control is instantaneous and requires no direct contact^4–6^. Therefore, nowadays energy conversion mechanism that requires light triggering has attracted considerable attention^7–9^. Ferroelectric photovoltaics represent one of the major breakthroughs in this direction which were investigated with respect to the generation of steady-state photocurrent and above band-gap photovoltage coupled to polarization^4^. Non-ferroelectric metal-coordination based dynamic molecular systems, on the other hand, might promise greater diversity for such objectives where physiochemical properties of the material can be orchestrated using external stimuli such as light or heat^10^. Therefore, they’re a perfect fit for developing next-generation energy conversion devices. Recent progress in the development of molecular pyroelectric crystals^11^, which undergo changes in the redox states and hence macroscopic polarization upon temperature variation, has paved the way to address the challenging aspect of realizing nano-scale energy storage and conversion in the same material (Fig. 1a). Generally in such systems, a thermally induced population change can occur between two electronic states associated with an intramolecular electron transfer, which is termed as valence tautomerism (VT)^12^. Notably, when a Co^3+^ ion and a tetraoxolene ligand are used as the electron acceptor and donor, respectively, VT can be easily tailored to occur with a spin transition at the Co site, in which the Co^3+^_LS_-based redox isomer (LS = low-spin) possesses a slightly lower electronic energy than the Co^2+^_HS_-based one (HS = high-spin)^13–15^. These two local minima are well separated on the potential energy surface with an energy barrier that hinders intramolecular electron transfer at low temperature, which prolongs the lifetime of the electron-transferred metastable excited state generated upon light irradiation (Fig. 1b). When such molecules crystallize in polar space groups (Fig. 1c), which prevent molecular dipoles from canceling each other due to symmetry restrictions, the higher polarization state (excited polarization state) of the crystals can be persistently trapped upon light excitation. This trapping of excited polarization state is responsible for energy storage inside the molecule that is cumulatively manifested over the entire crystal. Furthermore, during relaxation from the trapped metastable state due to the gradual alteration of macroscopic polarization with time (δ*P*/δ*t*), that stored energy inside the material release via physically detectable electric current (Fig. 1a). Hence, repeated electricity production in such system originates from electron transfer event of molecular origin upon light irradiation and does not need any electric field, conjunction, or transport layer fabrication. Also, it aspires for miniaturization due to its non-correlated material nature, thus illustrates a promising alternative to conventional photovoltaics.

Along this consideration, here we provided a proof-of-concept of photoenergy storage and conversion in a polar dinuclear metal complex [(Co(*RR*-cth))((Ga(*SS*-cth))(*μ*-dhbq)](PF_6_)_3,_ abbreviated as [CoGa] complex (dhbq = dihydroxybenzoquinone and cth = 5,5,7,12,12,14-hexamethyl-1,4,8,11-tetraazacyclotetradecane) where energy can be trapped at low temperature in terms of the above-mentioned light-induced VT (LIVT)^16–19^. The trapped energy is manifested as current release due to the change in polarization at the macroscopic scale. The Co complex exhibits electronic bistability where intramolecular charge transfer nominally involves the transfer of an electron from the dhbq^3-^- π^*^ orbital to the LS-Co^III^ centre to form a dhbq^2-^ and a HS-Co^II^ induced by visible light irradiations. The light-induced metastable state exhibits a long lifetime of more than 9 h (at 10 K) and an accelerated relaxation rate upon heating, affording a peak of current release at around 47 K. A thorough analysis including single crystal X-ray diffraction, temperature dependent X-ray absorption spectroscopy, infrared (IR) spectroscopy and density functional theory (DFT) calculations allows establishing a correlation of the molecular structure with macroscopic polarization in terms of the interconversion between two distinct electronic states [Co^3+^_LS_–dhbq^3−^–Ga^3+^] and [Co^2+^_HS_–dhbq^2−^–Ga^3+^].

## Results

### Synthesis and magnetic properties

**Fig. 2:**
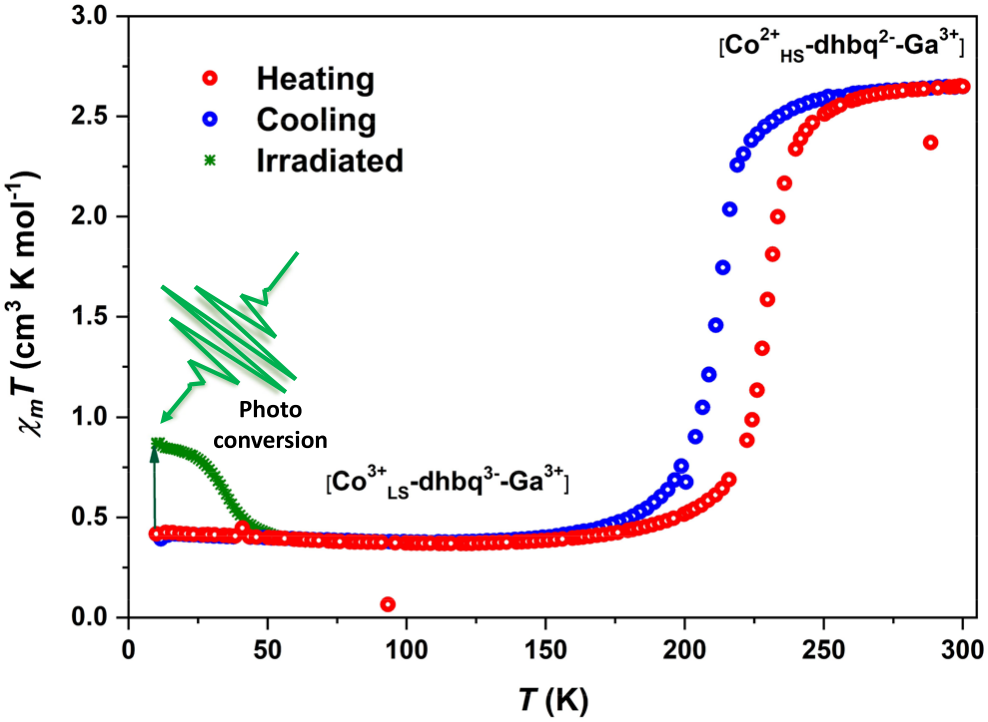
Thermal and light induced magnetic properties of the [CoGa] crystals. Temperature dependent *χ*_*m*_*T* product measured between 5 and 300 K in heating and cooling cycles under the temperature scan rate of 5 K min^−1^. The green dots represent light-induced (532 nm, 20 mW cm^−2^) valance tautomerism (LIVT) effect at low temperature. Scan rate after light-irradiation is 1 K min^−1^.

Polar crystals of the [CoGa] dinuclear metal complex were synthesized according to our previously reported methodology^20^. The selective formation of the heterometallic dinuclear complex [(Co(*RR*-cth))((Ga(*SS*-cth))(*μ*-dhbq)](PF_6_)_3_, **1(PF**_**6**_**)**_**3**_ was confirmed by electrospray ionization mass spectrometry (Supplementary Figs. 1, 2) and circular dichroism (CD) analyses. The enantiomer of **1(PF**_**6**_**)**_**3**_, [(Co(*SS*-cth))(Ga(*RR*-cth)(*μ*-dhbq)](PF_6_)_3_ was also obtained and its CD spectra was compared with **1(PF**_**6**_**)**_**3**_, displaying a mirror-image relationship in the visible light region (Supplementary Fig. 3), indicating the sheer stability of optically pure enantiomer in solution phase. A polycrystalline sample crystallized from the methanolic solution was subjected to direct current SQUID magnetometry measurements to confirm the VT transition under an external magnetic field of 0.5 T and temperature scan rate of 5 K min^−1^ (Fig. 2 and Supplementary Fig. 4). The bulk phase purity of the complex was confirmed by the well agreement of powder X-ray diffraction data with the simulated pattern obtained from X-ray crystal structure (Supplementary Fig. 5). The *χ*_*m*_*T* value (where *χ*_*m*_ is the molar magnetic susceptibility and *T* is the temperature) at 300 K was 2.64 cm^3^ K mol^−1^, which is close to that expected for a HS Co^II^ ion (S = 3/2) and a dhbq^2−^ ligand^20^. On cooling, an abrupt variation in *χ*_*m*_*T* was observed around *T*_*1/2*_ ↓ = 213 K, reaching 0.5 cm^3^ K mol^−1^ at 180 K, where *T*_*1/2*_↓ is defined as the transition temperature in the cooling mode. Upon further cooling down to 5 K, no significant change in *χ*_*m*_*T* value is observed and the value is consistent with the *S* = 1/2 of [Co^3+^_LS_–dhbq^3−^–Ga^3+^] state with a single unpaired electron of dhbq^3-^. On heating, an abrupt variation in *χ*_*m*_*T* was observed around *T*_*1/2*_ ↑ = 229 K and recovered to the original value at around 260 K, where *T*_*1/2*_↑ is defined as the transition temperature in the heating mode. The width of the thermal hysteresis loop was 16 K. Corresponding exothermic and endothermic peaks were also obtained by the differential scanning calorimetry (DSC) measurement (Supplementary Figs. 6, 7).

### Single-crystal structure

The single-crystal structures of **1(PF**_**6**_**)**_**3**_ were carefully analysed at low-temperature phase (150 and 200 K) and high-temperature phase (250 and 300 K) according to the magnetic measurement to characterize the structural change during the transition process (Supplementary Table 1). The obtained crystal structure is isomorphous with the dinuclear complexes of the same family, previously reported^20,21^. The discrete cationic molecule of **1(PF**_**6**_**)**_**3**_ consists of [*Δ*–Ga(*SS*-cth)] and [*Λ*–Co(*RR*-cth)] moieties that are bridged by a deprotonated dhbq ligand, forming an almost symmetrical molecular structure with a pseudo-inversion centre which crystallizes in the polar space group *P*2_1_ irrespective of the temperature. The bond lengths between the metal ion and N atoms of the tetraazamacrocyclic ligand are characteristic of the respective oxidation number and spin-state of the coordinated metal ion^11^. Thus, the observed bond lengths around the Co ions (Co–O, 1.89–1.90 Å; Co–N, 1.99–2.02 Å) and the Ga ions (Ga–O, 1.95 Å; Ga–N, 2.08–2.13 Å) at LT phase are consistent with typical Co^3+^_LS_ and Ga^3+^ (Supplementary Table 2). At 300 K, no significant change in the bond-length was observed around the Ga ions (Ga–O, 1.96 and 1.98 Å; Ga–N, 2.06–2.12 Å). In contrast, those involving the Co centre encounters elongation of bond-lengths (Co–O, 2.12 and 2.13 Å; Co–N, 2.11–2.15 Å) which is a clear signature of thermally induced VT process in the Co-dioxolene family^22^, and suggests the existence of the [Co^2+^_HS_–dhbq^2−^–Ga^3+^] electronic state at the HT regime (Supplementary Table 2 and Supplementary Fig. 8). These [Co–dhbq–Ga] moieties are alternatively arranged along with the crystallographic *b*-axis maintaining the same angle between Co→Ga vector and the crystallographic *b*-axis, which is *ca*. 21°. Hence, the change in the molecular dipole moment via directional electron transfer between the Co ion and the redox-active ligand can lead to the polarization change at the single-crystal level. DFT calculations were employed to determine the direction and magnitude of the dipole moments of the distinct states dominating the HT and LT regimes (Supplementary Fig. 9). In the HT phase, spin-quartet [Co^2+^_HS_–dhbq^2−^–Ga^3+^] state possesses a permanent electric dipole moment of 9.39 Debye from the Co ion to the Ga ion, whereas the spin-doublet state turned to be nearly nonpolar with a dipole moment of 0.05 Debye at LT along the same direction. By projecting the change in molecular dipole moments to the polar axis, the net polarization change between 150 and 300 K was estimated to be 2.05 μC cm^−2^.

### Spectroscopic study

**Fig. 3:**
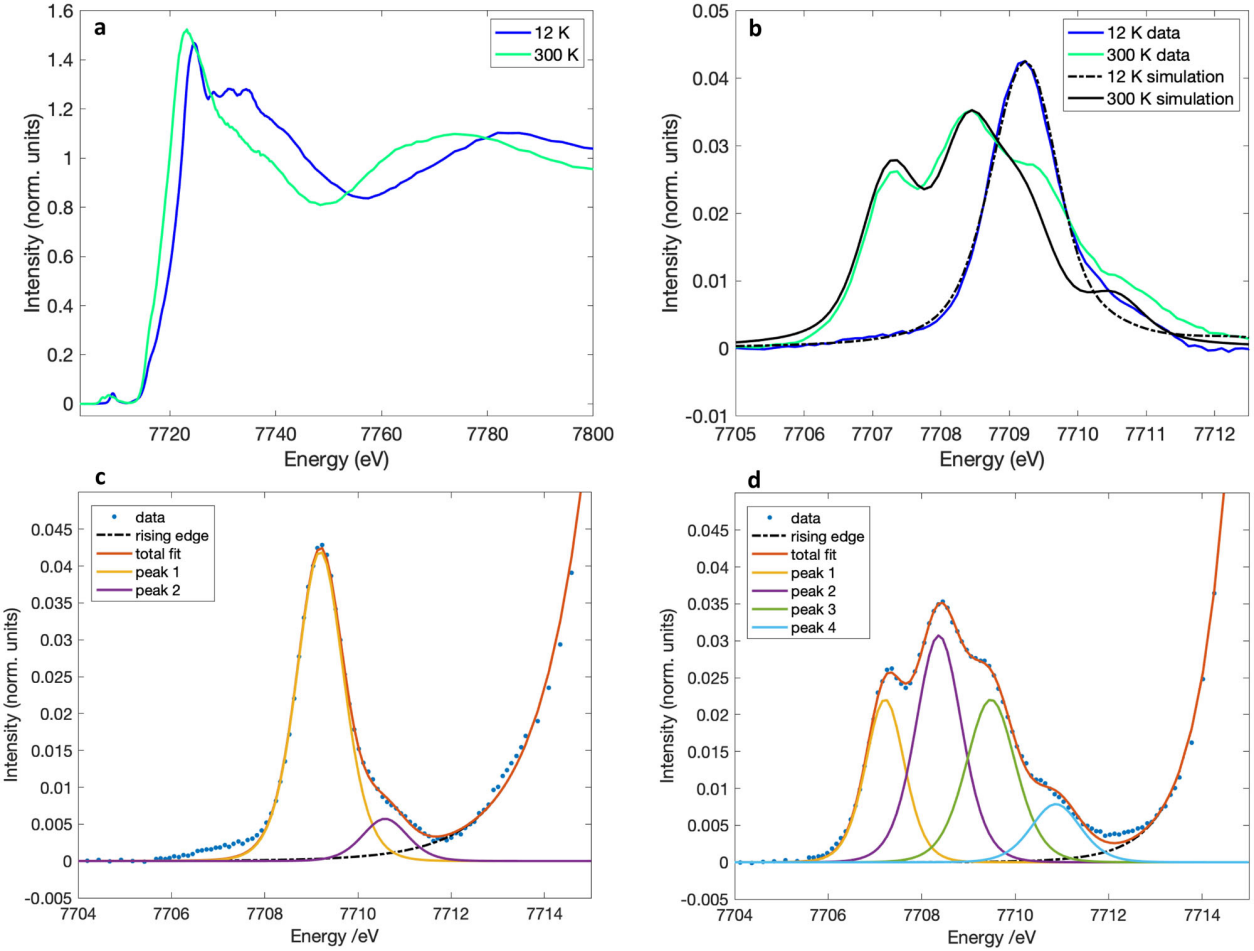
High energy-resolution fluorescence detected X-ray absorption near edge structure (HERFD-XANES) of [CoGa] crystals. **a** Temperature-dependent Co K-edge HERFD-XANES (**b**) Comparison of Co HERFD-XANES pre-edges measured at low temperature (12 K) and high temperature (300 K). The black lines represent ligand-field multiplet simulations (**c**) Co HERFD-XANES with deconvolution of peaks for the 12 K spectrum. **d** Co HERFD-XANES with deconvolution of peaks for the 300 K spectrum.

**Fig. 4:**
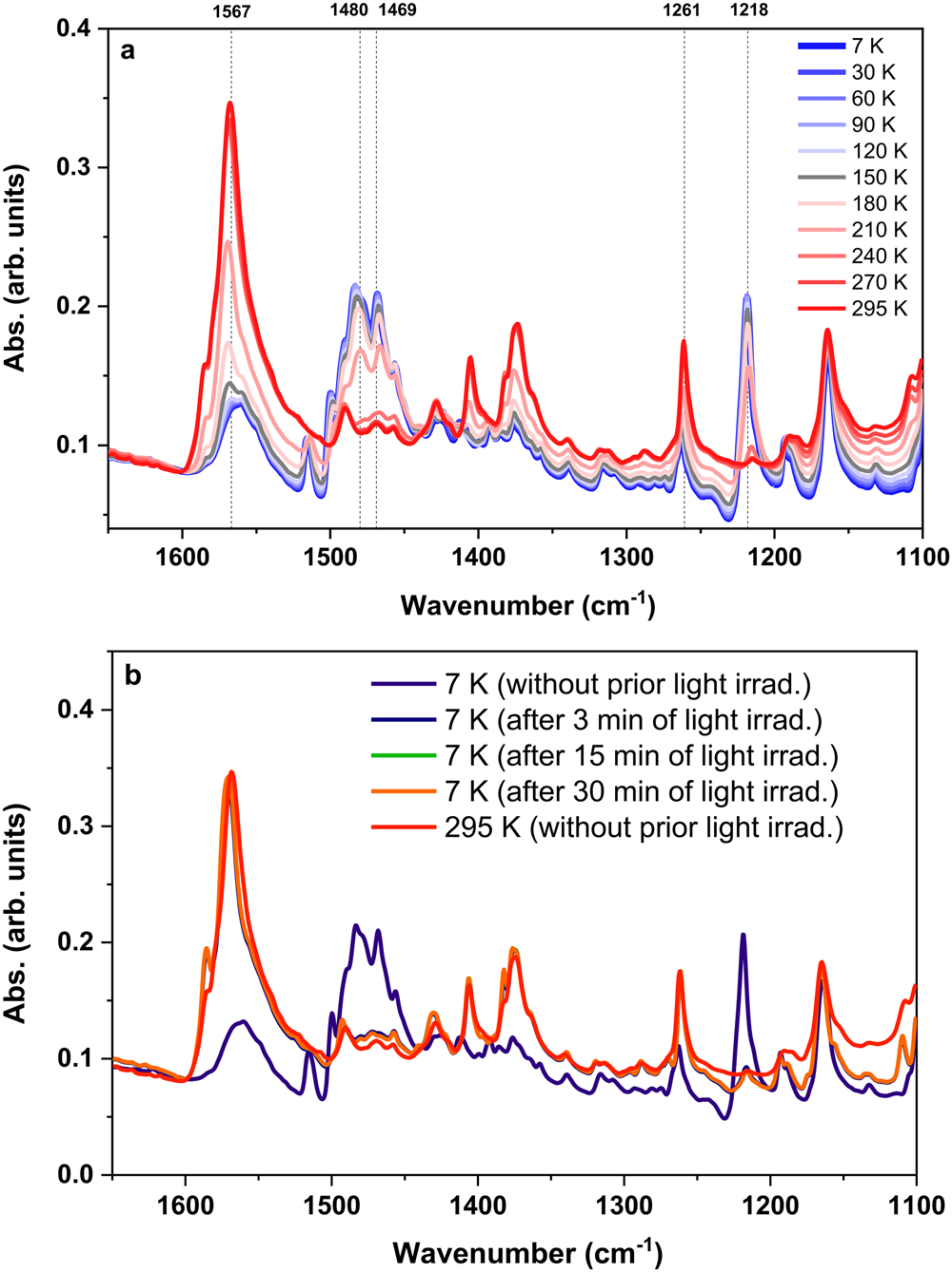
IR spectroscopy of [CoGa] complex. **a** Temperature dependent IR spectra between 295 K and 7 K. Dashed (or dotted) black lines indicate peak positions of the marker bands mentioned in the text. **b** Composition of IR-spectra before and after photo irradiation (530 nm, 15 mW cm^–2^) recorded at 7 K.

To confirm the change in the valence of the Co site during the transition process, Co K-edge high-energy resolution fluorescence detected X-ray near edge absorption spectroscopy (HERFD-XANES) measurements were performed at various temperatures^23–25^. A clear shift in the energy of the absorption edge threshold and a change in the fine structure were consistent with the valence change and spin transition at the Co site (Fig. 3). An analysis of the Co K pre-edge provided greater insight into the 3d electronic structure. At room temperature (300 K), the pre-edge absorption was resolved in four 1 s → 3d quadrupole-allowed transitions that can be assigned assuming an approximately octahedral coordination symmetry ^4^T_1_(t_2g_^5^e_g_^2^) for the ground state of Co^2+^_HS_. The promotion of a 1 s electron into the 3d manifold gave the four excited states ^3^A_2_(t_2g_^6^e_g_^2^), ^3^T_2_(t_2g_^5^e_g_^3^), ^3^T_1_(t_2g_^5^e_g_^3^), and a transition-forbidden two-electron transition configuration ^3^T_1_(t_2g_^4^e_g_^4^). However, since the two ^3^T_1_ states are allowed to mix by symmetry both ^3^T_1_ states have one electron allowed (t_2g_^5^e_g_^3^) character. The measured energy splitting between ^3^A_2_(t_2g_^6^e_g_^2^) and ^3^T_2_(t_2g_^5^e_g_^3^) states, at 7707.2 and 7708.35 eV respectively, relate to the 3d ligand field splitting. The measured energy splitting between the two ^3^T_1_ states, at 7709.5 and 7710.8 eV respectively, relate to the Racah interelectronic repulsion. A ligand field multiplet simulation^26,27^ with a 10Dq parameter of 1.15 eV and an interelectronic repulsion slater reduction factor of 0.7 confirms these assignments (Supplementary note 1), reproducing the relative intensities and energies of the experimental spectrum (black line, Fig. 3b). Upon cooling down to 12 K, only one pre-edge peak remains, which can be attributed to the excitation from the ^1^A_1g_(t_2g_^6^e_g_^0^) ground state of Co^3+^_LS_ to a ^2^E(t_2g_^6^e_g_^1^) excited state, further corroborating the conclusion that valence change occurs on the Co site. IR spectroscopy provided complementary information about the change in the electronic structure of the bridging ligand. Specifically, the strong bands associated with CO stretching modes in the range between 1650 and 1100 cm^−1^, which are sensitive to the electronic state of the tetraoxolene ligand, were used to investigate the corresponding transition behaviour (Fig. 4 and Supplementary Fig. 10). Thus, the spectrum at low temperature was consistent with the dhbq^3−^ character of tetraoxolene, showing peaks characteristic of the CO stretching vibrations of dhbq^3−^ at approximately 1480, 1469, and 1218 cm^−1^, whereas at high temperature the appearance of peaks at approximately 1567 and 1261 cm^−1^, which correspond to the CO stretching vibrations of dhbq^2−^^21^, suggests the existence of a dhbq^2−^ species. These results clearly confirm the thermal bistability of the [CoGa] crystals (Fig. 4a).

The occurrence of thermally induced VT also induced a change in the absorption of electronic transitions (Supplementary Fig. 11). Distinct peaks were observed at around 485 and 520 nm in UV-vis absorption spectra which can be attributed to the π → π* band of dhbq^3-^ based on the charge density difference between the ground and excited states (Supplementary Fig. 12). The intensity of these peaks gradually decreased with increasing the temperature up to 300 K.

### Optical control on energy conversion

**Fig. 5:**
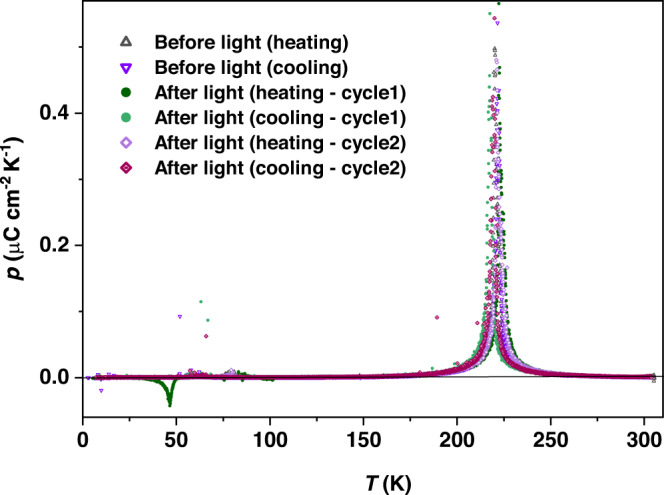
Pyroelectric properties of [CoGa] crystals before and after the light irradiation. Pyroelectric coefficient (*p*) was measured on a single crystal of **1(PF**_**6**_**)**_**3**_ consisting of three consecutive heating and cooling cycles between 5 and 310 K where the 1st cycle was measured before the light irradiation and following next two cycles were measured after a prolonged green light irradiation upon the sample. Temperature sweep rate for these pyroelectric measurements is 5 K min^−1^. Note that pyroelectric coefficient at round 47 K after light irradiation (heating—cycle 1) is negative, meaning that the direction of electric current is opposite to that at around 222 K on heating mode.

**Fig. 6:**
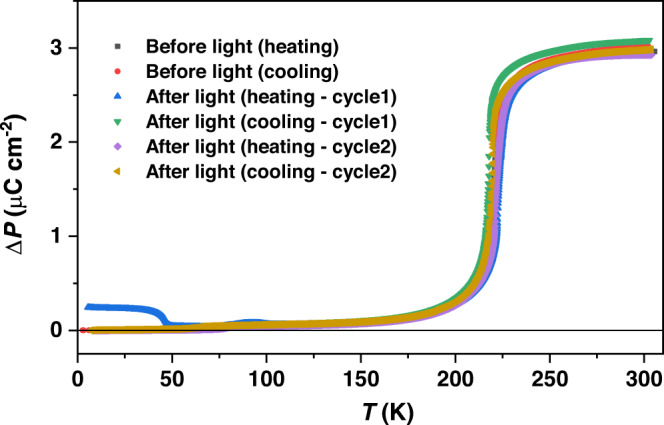
Macroscopic polarization changes before and after light irradiation between 5 and 310 K recorded in heating and cooling mode. Temperature sweep rate for these pyroelectric measurements is 5 K min^−1^.

As mentioned above, the molecular level dipole moment change in the VT transition can be promoted to the single crystal level due to the polar crystal structure of **1(PF**_**6**_**)**_**3**_ To determine the magnitude of the polarization change, the pyroelectric property was measured between 5 and 300 K. Sharp peaks of the pyroelectric coefficient (*p* = δ*P*/δ*T*) were clearly observed at around 222 K where the electron transfer process occurs (Fig. 5). By integrating the current over this temperature domain the change in macroscopic polarization was obtained, which was almost zero upto the transition point, but increased suddenly during the transition up to 2.9 μC cm^−2^ (Fig. 6). This value is higher than the theoretically estimated value of 2.05 μC cm^−2^ possibly due to the secondary pyroelectric effect (Supplementary notes 2,  3).

Notably, the coupling of the spin transition with the electron transfer behavior in Co VT systems frequently leads to light-induced VT at low temperatures, enabling the trapping of the electron-transferred metastable state^16^. Therefore, in our system, the LIVT phenomenon could directly afford light-induced polarization switching, converting light energy into electrical energy stored in the regular molecular arrays (single crystals). To evaluate such a scenario, the powder sample attached to a CaF_2_ plate was irradiated with a 530-nm green LED light source to excite the π → π* band of dhbq^3−^, and the corresponding light-induced IR absorption spectra were recorded. During light illumination at 7 K, the IR spectrum changed within 3 min of irradiation time and became more similar to that observed at 295 K without irradiation (Fig. 4), suggesting the presence of the trapped electron-transferred metastable electronic state [Co^2+^_HS_–dhbq^2−^–Ga^3+^]. After trapping the metastable state at low temperature, the light was turned off and the IR spectra were recorded every 30 min. Supplementary Fig. 13 demonstrates that the lifetime of the metastable state was sufficiently long because the majority of the molecules were still in dhbq^2−^ state even after 120 min of turning off the light. In addition, since the metastable state quickly relaxed to the ground state at temperatures above 60 K, no HS species could be detected by IR spectroscopy (Supplementary Fig. 14).

To quantitatively describe the LIVT behavior, a polycrystalline [CoGa] sample was irradiated with a green light (532 nm CW laser) at 5 K. An increase in the magnetization value was observed. Figure 2 displays the distinct light-induced magnetization change behavior. When the irradiation was stopped, the *χ*_*m*_*T* of the photoproduct was measured as a function of time to investigate the relaxation kinetics of the photoinduced metastable state. For each run, the crystalline sample was cooled to the set temperature and illuminated until the magnetization value reached saturation. Fitting with the stretched exponential law yielded a relaxation time of around 9 h at 10 K (Supplementary Fig. 15), supporting the IR result that suggested the existence of a long-lived metastable state for the [CoGa] system, which can be attributed to the large change in the metal–ligand bond length preventing the fast relaxation from [Co^2+^_HS_–dhbq^2−^–Ga^3+^] to [Co^3+^_LS_–dhbq^3−^–Ga^3+^] (Supplementary Note 4). However, the trapping of the metastable state and the relaxation process are very similar to those attributed to the LIESST effects^28^. The temperature dependence of the *χ*_*m*_*T* values was evaluated by increasing the temperature between 5 and 300 K (Fig. 2). The saturated *χ*_*m*_*T* value of 0.86 cm^3^ K mol^−1^ at 10 K was maintained up to approximately 25 K and then relaxed to the initial value at approximately 40 K (*T*_LIVT_), which indicates a relevant photoconversion (20%) of the [Co^3+^_LS_–dhbq^3−^–Ga^3+^] state.

Taken together, these results suggest that macroscopic polarization switching at the single-crystal level can be induced via the LIVT process in this polar system with directional electron transfer. Therefore, the current release was measured on a block-shaped face-indexed single crystal of **1(PF**_**6**_**)**_**3**_ sandwiched between two silver electrodes^29^. The sample was preliminarily illuminated by green light with an intensity of 20 mW cm^−2^ for one hour, and then the light was turned off before starting the measurement. The experimental current generation was measured with a Keithley 6517B electrometer while heating the sample from 5 K to room temperature at a scan rate of 5 K min^−1^ (Fig. 5 and Supplementary Fig. 16). Light-induced current at low temperature (*T*_LIVT_) and thermally induced current at high temperature (*T*_1/2_) were detected, which is in good agreement with the change in the magnetic properties observed in the whole temperature range (Supplementary Figs. 16, 17). Between 5 and 60 K, a sharp peak was observed at *T*_LIVT_ up to *i*_p_ = *pA*(δ*T/*δ*t*) ~ 12 pA (where *A* is the area of the measured electrode surface) with a net polarization change (Δ*P*) of around 0.20 μC cm^−2^, which can be attributed to the thermally activated relaxation from the light-induced metastable state to the ground state (Fig. 6 and Supplementary Fig. 18). The peak temperature in pyroelectric current is slightly higher than that in d(*χ*_m_*T*)/d*T* vs. *T* plots. The observed difference between the magnetic and pyroelectric measurements could be attributed to the different experimental conditions. The scan rate of magnetic and pyroelectric measurements is 1 K min^−1^ and 5 K min^−1^, respectively. Additionally, magnetic measurements were conducted on a crystalline powder sample, while a well-shaped single crystal was used for the pyroelectric measurements.

**Fig. 7:**
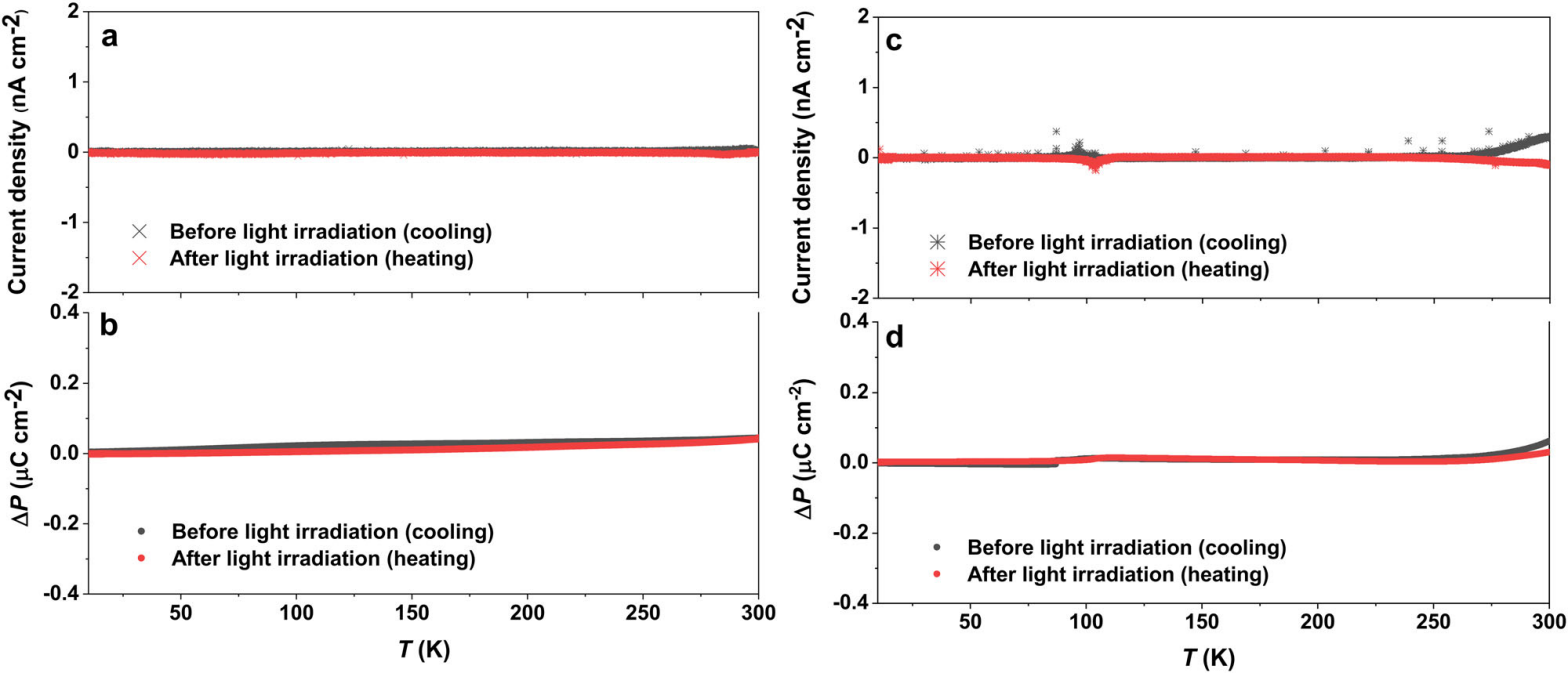
Optically induced current response of reference complexes before and after the light irradiation. **a** Absence of photoinduced current release during the temperature range of 10–300 K for [CoCo](PF_6_)_3_. **b** Absence of light-induced polarization changes for [CoCo](PF_6_)_3_. **c** Absence of photoinduced current release for [CrCo](PF_6_)_3_. **d** Absence of light-induced polarization changes for [CrCo](PF_6_)_3_. Temperature sweep rate for these pyroelectric measurements is 5 K min^−1^.

To investigate whether the energy conversion from light energy to electrical energy in the molecular system is inherent to polar structures exhibiting LIVT phenomenon, the current responses of two reference complexes were compared after the light irradiation (Supplementary Table 3). First, the current was measured on a complex crystallizing on the nonpolar space group *P*2_1_/*c*, i.e., [Co(*RR*-cth)–dhbq–Co(*SS*-cth)](PF_6_)_3_, which exhibits a similar molecular packing to that of **1(PF**_**6**_**)**_**3**_ and LIVT phenomenon^18^. It has been reported VT transition temperature is about 175 K and the photoinduced metastable state relaxes back to the ground state below around 60 K^18^. No current release was detected because the inversion symmetry at the crystal level canceled the polarization (Fig. 7a, b). Second, a similar measurement was conducted on a [Cr(*SS*-cth)–dhbq–Co(*RR*-cth)](PF_6_)_3_ complex with VT transition temperature of approximately 360 K (Supplementary Fig. 19), which crystallizes in the isostructural polar space group *P*2_1_ and exhibits only a small LIVT behavior. This also resulted in the absence (below the detection limit) of current release (Fig. 7c, d)^20^. These comparative results clearly demonstrate the singularity of the optical control upon energy conversion of this polar molecular material exhibiting LIVT.

## Discussion

The direct observation of optical control upon energy conversion and storage in a polar molecular [CoGa] crystal is demonstrated. Unlike traditional ferroelectric photovoltaics, the energy conversion in our system has an intramolecular origin, namely, the directional electron transfer in a polar crystal resulting in a metastable polarization state. In ferroelectrics, spontaneous symmetry breaking requires collective lattice modes. But our noncorrelated molecular approach circumvents these drawbacks by offering flexibility and designability while not requiring a poling field to enhance the internal field due to its non-ferroelectric nature^8^. The present molecular design based on an electron-transferred metastable state and a polar space group can be considered generic and extendable to even pure organic systems, one may use the crystal structure databases for mining out potential candidates with desired properties or modifying with relevant functionalities; such as the working temperature of the photoinduced current release (relaxation temperature from the light-induced metastable state to ground state) can be tuned based on different chemical environment around the metal centre. A longer metal-ligand bridging can also be employed to enhance the polarization change as well as more energy restoration. Further research along this line is underway to explore more exotic and application oriented new properties in the arena of modern material science.

## Methods

### Synthesis

All solvents and reagents were used as received from FUJIFILM Wako Pure Chemical Co., Tokyo Chemical Industry Co. and Sigma-Aldrich. Racemic-cth and the enantiopure ligands (*RR*-cth and *SS*-cth) (cth = 5,5,7,12,12,14-hexamethyl−1,4,8,11-tetraazacyclotetradecane) were prepared according to literature procedures^30,31^.

Preparation of racemic-cth monohydrate: A total of 111.4 g of 60% perchloric acid (0.66 mol) was slowly added into the acetone solution (1 L) of ethylenediamine (40 g, 0.67 mol) in an ice bath and was stirred for 6 h. The white solid of 5, 7, 7,12, 14, 14-hexamethyl−1, 4, 8, 11- tetraazacyclotetradeca-4, 11-diene diperchlorate was collected by filtration and was washed with acetone (142.8 g, yield = 89.9%). Then, the white crude product (100 g, 0.21 mol) was dissolved in methanol (600 mL) maintaining the temperature 0–5 °C. The solid of NaBH_4_ (19 g, 0.63 mol) and NaOH (16.5 g, 0.42 mol) were added alternately in small portions to the solution. After the completion of the addition, the mixture was stirred at room temperature for 1 h, followed by heating the solution and boiling it for 15 min. Then the solution cooled down to room temperature, an aqueous solution of NaOH (50 g in 1 L water) was added, and the solution was stirred for another 2 h. White solid (mixture of meso- and racemic cth) was obtained by filtration and dried in air for one day. Then, the product was dissolved in 600 mL of methanol. Water (400 mL) was added into the methanol solution and stirred for 2 h; the precipitate of meso-cth was formed gradually and was removed by filtration. Another 200 mL of water was added to the solution, and the precipitate formed after stirring for 1.5 h was removed again by filtration. The solution was evaporated to near dryness on a rotatory evaporator, and the white solid product of racemic cth monohydrate was collected by filtration and washed with a small amount of cold water and dried in air (20.4 g, yield = 32.4%).

Optical resolution of cth: To a solution of nickel diacetate tetrahydrate (7.46 g, 30 mmol) in 125 mL of methanol is added *rac*-cth monohydrate (7.56 g, 25 mmol). The solution was stirred and heated at 60° Celsius for 1 h and a blue-violet solution is obtained. sodium perchlorate (6.12 g, 50 mmol) is added to the solution and stirred for 30 min at room temperature and a blue-violet solid of [Ni(AcO)(*rac*-cth)]ClO_4_ is precipitated which was collected by filtration and dried under vacuum. 11 g of [Ni(AcO)(*rac*-cth)]ClO_4_ is dissolved in 100 mL water and 4.17 g of 60% HClO_4_ was added dropwise to obtain yellow precipitate of [Ni(*rac*-cth)](ClO_4_). Addition of solid tetra-n-butylammonium bromide (7.2 g, 22.4 mmol) to the purple solution of [Ni(*rac*-cth)](ClO_4_) (4.0 g, 7.4 mmol) in 160 mL of acetonitrile obtained green precipitate of the bromide salt [NiBr_2_(*rac*-cth)]. 4 g of bromide salt were dissolved in 160 mL of water at room temperature and sodium d-tartrate dihydrate (0.5 g, 2.2 mmol) were added by dissolving in water. Color of the solution turned greenish brown gradually. After 30 min, sodium perchlorate (0.49 g, 4.0 mmol) dissolved in a minimum amount of water is added to this solution and the solution was chilled in an ice-bath for 1 h. The blue-violet precipitate is then filtered and washed with chilled water, EtOH and ether. This blue-violet precipitate is [{Ni(*SS*-cth)}_2_(d-tart)(H_2_O)](ClO_4_)_2_•2H_2_O(compound A). The filtrate contains *RR*-enantiomer and to this solution added 1.0 g of sodium oxalate and 1.0 g of sodium perchlorate. That solution was then basified to pH ~10 with aqueous sodium hydroxide to obtain a pale blue product ([{Ni^II^(*rac*-cth)}_2_ox](ClO_4_)_2_), which was filtered out. The pH of the blue-filtrate was adjusted to ~1 by dropwise addition of 60% perchloric acid in an cold ice-bath to yield a blue-violet product which is optically pure [(Ni(*RR*-cth))_2_(ox)](ClO_4_)_2_ (compound B). Compound A (0.85 g, 0.78 mmol) was dissolved in a mixture of 20 mL water and 10 mL EtOH. NaOH(1.4 g, 35 mmol) and 0.51 g of NaCN(0.51 g, 10.4 mmol) were then added to the solution and refluxed for 3 h during which the solution turned to yellow from purple. Ethanol was then boiled off from the solution with an oil bath set at 90 °C. After cooling the reaction solution to room temperature, the free *SS*-cth was extracted with diethyl ether (20 mLx3). The ether layer was washed 3-4 times with water, dried with Na_2_SO_4_ and then removed solvent by rotary evaporator to give *SS*-cth(0.41 g, yield = 92%) as white powder. A similar treatment on compound B(0.77 g, 0.78 mmol) yielded *RR*-cth(0.39 g, yield = 87%).

All reactions were conducted under a dry N_2_ atmosphere. For the synthesis of [GaCl_2_(*SS*-cth)]Cl, a solution of anhydrous GaCl_3_ (1.0 g, 6.0 mmol) in dehydrated DMF (30 mL) was boiled for 2 h. *SS*-cth (1.7 g, 6.0 mmol) in dehydrated DMF(5 mL) was added to the hot solution resulting the deposition of a white powder. The final product was collected from the cooled solution after filtration and washing with 2-propanol and Et_2_O (1.7 g, yield = 62%).

[Co(AcO)(*RR*-cth)](PF_6_) was synthesized according to literature. A mixture of Co(AcO)_2_·4H_2_O (747 mg, 3.0 mmol) and *RR*-cth (850 mg, 3.0 mmol) in EtOH (12 mL) was heated under a N_2_ atmosphere. After stirring at 65 °C for 15 min, solid NH_4_PF_6_ (540 mg, 3.3 mmol) was added to the solution. [Co(AcO)(*RR*-cth)](PF_6_) then gradually precipitated within 30 min. The reaction mixture was then cooled in an ice-bath and a precipitate was collected by filtration and washed with cold EtOH followed by Et_2_O. [Co(AcO)(*RR*-cth)](PF_6_) was obtained as slightly pink crystalline solid (1.16 g, yield = 71%). Anal. C_18_H_39_N_4_O_2_F_6_PCo (547.42) Calcd. C: 39.49, H: 7.18, N: 10.23; found C: 39.45, H: 7.17, N: 10.26.

### Synthesis of [(Co(*RR*-cth))(Ga(*SS*-cth))(*μ*-dhbq)](PF_6_)_3_

A solution of 3,5-dihydroxy-1,4-benzoquinone (H_2_dhbq; 140 mg, 1.0 mmol) and triethylamine (202 mg, 2.0 mmol) in MeOH (50 mL) was bubbled with dry N_2_ gas for 5 min to remove oxygen. Subsequently, solid [Co(AcO)(*RR*-cth)](PF_6_) (544 mg, 1.0 mmol) and [GaCl_2_(*SS*-cth)]Cl (460 mg, 1.0 mmol) were added to the solution, and the reaction mixture was refluxed for 2 h. A hot solution of KPF_6_ (276 mg, 1.5 mmol) in H_2_O (30 mL) was added to this reddish solution. The mixture was slowly cooled and then kept at room temperature. After 24 h, a brown precipitate of [(Co(*RR*-cth))(Ga(*SS*-cth))(*μ*-dhbq)](PF_6_)_2_Cl was collected by filtration and washed with cold H_2_O (781 mg, yield = 67%). Solid AgPF_6_ (76 mg, 0.3 mmol) and H_2_O (1 mL) were added to a solution of [(Co(*RR*-cth))(Ga(*SS*-cth))(*μ*-dhbq)](PF_6_)_2_Cl (335 mg, 0.3 mmol) dissolved in MeCN (60 mL). After stirring for 10 min, the mixture was filtered to remove AgCl. The filtrate was evaporated to dryness under reduced pressure, and crude **1(PF**_**6**_**)**_**3**_ was collected as a brown solid and washed with a small amount of H_2_O. The crude product was recrystallized from a mixed solvent of several drops of MeCN and H_2_O/MeOH to afford dark-red crystals suitable for structural analysis (952 mg, yield = 75%). Elemental analysis calcd. for C_38_H_74_N_8_O_4_F_18_P_3_GaCo: C: 36.08, H: 5.85, N: 8.86, Ga: 5.48, Co: 4.64; found: C: 35.82, H: 5.87, N: 8.84, Ga: 5.42, Co: 4.69; ESI MS (Supplementary Figs. 1,  2): *m/z* = 1124 [**1**(PF_6_)_2_]^+^ (calcd. for C_38_H_74_N_8_O_4_F_12_P_2_GaCo: 1124).

### Magnetic property

Magnetic susceptibility measurements were performed on a quantum design SQUID magnetometer (MPMS-5S) under a 5000 Oe field, with a sweeping rate of 5 K min^−1^ ^32^. The measurement samples were prepared by encapsulating well-grinded microcrystal sample of **1(PF**_**6**_**)**_**3**_ into a gelatin capsule, which was fixed on a sample rod with a plastic straw. Photomagnetic measurements were performed on the powdered sample attached to transparent tape. A Nd-YAG laser (HCP/GLMP-0200A, 532 nm, cw, 200 mW) was used for photoexcitation. The sample was illuminated for an hour with the cooling valve open to maintain the temperature at 5 K. All measurements were performed with the vent valve set to open mode.

### Pyroelectric measurements

The pyroelectric current data along the crystallographic *b*-axis was collected upon sweeping the sample temperature at a rate of 5 K min^−1^ under zero electric field with Keithley 6517B electrometer (continuous temperature ramping technique)^33^. The single-crystal sample was sandwiched by silver and carbon paste on its (010) and (0–10) surfaces to determine the direction of the pyroelectric current. Data were also recorded on a crystal piece sandwiched by two silver electrodes. All the pyroelectric and light energy conversion data of [CoGa] sample were measured upon the same piece of single-crystal with the vent valve set to open mode^29^. The measurement temperature was restricted between 5 and 310 K under a helium gas flow inside a Quantum Design MPMS-XL chamber. The data recording was started when the background current was below 0.04 pA to ensure the reliability of the current recorded. The short-circuit current at low temperature was measured by irradiating green light from the same laser used in the LIVT measurement.

### IR Spectroscopy

The IR spectra of crystalline [CoGa] complex at variable temperature and at 7 K before and after light (530 nm) irradiation were recorded using an FT-IR spectrophotometer (FT/IR-Vertex 70, Bruker) equipped with a closed-cycle helium refrigerator cryostat (Cryotec, Daikin industries, Ltd.). The ground-powdered samples were held between grained and flat CaF_2_ plates. A green LED (M530L4, Thorlabs Inc.) emitting 530 nm radiation was used as the illumination light source. The LED wavelength was 530 nm, and the power density was 15 mW cm^–2^ at the sample point. Temperature dependent baseline shift is removed by adjusting offset of each observed spectrum prior to the presentation.

## Supplementary information

Supplementary Information

## Data Availability

The supplementary crystal data can be obtained free of charge from the Cambridge Crystallographic Data Centre (www.ccdc.cam.ac.uk/data_request/cif) using identifiers CCDC 2149695-2149698. Source data are provided with this paper. All other data are available from the corresponding author upon request. Supplementary Information is available in the online version of the paper. Source data are provided with this paper.

## Source data

Source Data
