# Supplementary material for: Energy conversion and storage via photoinduced polarization change in non-ferroelectric molecular [CoGa] crystals
Source: Nat Commun. 2023 Jun 9;14:3394. doi: 10.1038/s41467-023-39127-8 (PMC10256679; doi:10.1038/s41467-023-39127-8)
Supplement: Supplementary file 1 — Supplementary Information [file 41467_2023_39127_MOESM1_ESM.pdf]

Supplementary Information

Energy conversion and storage via photoinduced polarization change in non-ferroelectric molecular [CoGa] crystals

Pritam Sadhukhan[1], Shu-Qi Wu[1], Shinji Kanegawa[1,*], Sheng-Qun Su[1], Xiaopeng Zhang[1], Takumi Nakanishi[1], Jeremy Ian Long[1], Kaige Gao[2], Rintaro Shimada[3], Hajime Okajima[3], Akira Sakamoto[3], Joy G. Chiappella[4,5], Myron S. Huzan[4,5], Thomas Kroll[6], Dimonthenis Sokaras[6], Michael L. Baker[4,5], Osamu Sato[1,*]

[1]Institute for Materials Chemistry and Engineering & IRCCS, Kyushu University, 744 Motooka, Nishi-ku, Fukuoka 819-0395, Japan.

[2]College of Physical Science and Technology, Yangzhou University, Jiangsu 225009, P. R. China.

[3]Graduate School of Science and Engineering, Aoyama Gakuin University, 5-10-1 Fuchinobe, Chuo-ku, Sagamihara, Kanagawa 252-5258, Japan.

[4]The Department of Chemistry, The University of Manchester, Manchester M13 9PL, UK.

[5]The Department of Chemistry, The University of Manchester at Harwell, Didcot OX11 0FA, UK.

[6]Stanford Synchrotron Radiation Lightsource, SLAC National Accelerator Laboratory, Stanford University, Menlo Park, California 94025, United States.

Correspondence and requests for materials should be addressed to S. K. (email: kanegawa@cm.kyushu-u.ac.jp) or to O. S. (email: sato@cm.kyushu-u.ac.jp).

X-ray structure Determination. A block shape single crystal was coated with an oil-based cryoprotectant and mounted on nylon loop. Diffraction data were collected at 150, 200, 250 and 300 K with the same crystal of **1(PF$_6$)$_3$** under a cold nitrogen gas-stream on a Rigaku FR-E+ diffractometer equipped with a HyPix-6000 area detector, using multi-layer mirror monochromated Mo-Kα radiation (λ = 0.71073 Å). The structures were solved by a direct method and refined *via* full-matrix least-squares on F^2 using the SHELX program[1] implemented in the OLEX2 program[2] with anisotropic thermal parameters for all non-hydrogen atoms (Supplementary Figs. 20, 21, 22, 23 and Supplementary Note 5). The hydrogen atoms were geometrically added and refined by the riding model. Room-temperature powder X-ray diffraction patterns were recorded on a Rigaku-TTR diffractometer to examine crystal purity.

X-ray Spectroscopy. X-ray spectroscopy measurements were performed at the Stanford Synchrotron Radiation Lightsource (SSRL), operated at 3 GeV with an electron beam current of 500 mA. The measured samples were maintained between 12 K and 300 K using an Oxford Instruments CF1208 continuous flow liquid helium cryostat. Orientation averaged powder samples were mixed in boron nitride to alleviate self-absorption effects. Co high energy resolution fluorescence detected (HERFD) measurements were performed at the undulator beamline 15-2. A double-crystal monochromator equipped with Si(311) crystals was used to select the energy with a resolution (ΔE/E) of ~10^{-5} and two Rh-coated Kirkpatrick-Baez mirrors set a 3.5 mrad delivered a 45-µm (v) x 800-µm (h) X-ray beam to the sample position. Monochromator energy calibration was performed using the first inflection point of an Co foil set to 7708.9 eV. Seven Si(531) crystals arranged in a 1m Rowland geometry were used to select the X-ray emission energy at a single element silicon drift detector. The emitted beam path was enclosed by a He-filled bag to reduce

the signal attenuation. To avoid radiation damage, a sample stage that is equipped with motors to allow for horizontal and vertical movement for multiple sampling positions was employed. Radiation damage was carefully monitored through consecutive scans at the same spot and the exposure time/dose per irradiated spot was kept well below the levels required for noticeable beam-induced spectral alterations. The spin transition observed in variable temperature was confirmed to be reversible through a complete heating cooling cycle. For each HERFD spectrum the emission detector energy was set to the maximum of the Co Kα1 emission line, 6929.75 eV for Co^{III}_{LS} and 6930.1 eV for Co^{II}_{HS}.. Pre-edge peak fitting for HERFD results were performed using a Pearson VII line shapes with a fixed 50:50 ratio of Lorentzian to Gaussian functions where the energy positions, the full width half maximum and the peak heights were optimised using a non-linear least squares fitting. Octahedral symmetry ligand field multiplet simulations of the Co K pre-edge were performed using the Crispy[3] interface for Quanty.[4] Multiplet effects were described by the Slater–Condon–Shortley parameters (F2 and F4) and were reduced to 70% of the Hartree–Fock calculated values in both initial and final states to account for the over-estimation of electron–electron repulsion found for the free ion and the nephelauxetic effects.

Density functional theory (DFT) calculations

DFT calculations for the [CoGa] dinuclear complex in the singlet, triplet, quintet and septet spin states were performed by unrestricted DFT (UDFT) implemented in the Gaussian 09 program package (Revision E.01).[5] To remove structural inaccuracies from incomplete spin transitions mentioned above, the DFT-optimized structures were used to compare the relative energies of different spin states and their corresponding vibrational spectra. For the Fe and Co atoms, the (14s9p5d)/[9s5p3d] primitive set of the Wachters–Hay basis[6,7] with one polarization f-function was used, and for the H, C, N, and O atoms, the 6-311+G** basis set was used.[8] Whereas the

relative energies of different spin states play a notable role in a multi-spin system, it is well known that DFT methods are less accurate at predicting small energy gaps between different spin states. The B3LYP functional[9] is often used to predict the geometry and spectroscopic properties of systems containing transition metals; however, this tends to overestimate the stability of the high-spin state.[10] Here we used a re-parameterized version of B3LYP functional, B3LYP*, for the calculations for the estimation of accurate energy differences between different spin state.[11] Vibrational analyzes were performed for all spin states to ensure that no imaginary frequencies existed for all optimized geometries. The vertical transition energies from the optimized geometry were investigated by time-dependent DFT method with the same basis sets[12,13]. The first 50 excitations were calculated to cover the range of interest, and the assignments of the observed absorption bands from the UV-vis spectra were based on the calculated configuration-interaction expansion coefficients and charge density difference.[14]

ESI mass spectrometry. Samples in solution were prepared by dissolving complexes in MeCN and acetone. All data were collected using a JMS-T100CS (JEOL) spectrometer in ESI+ mode at room temperature.

Circular dichroism (CD) measurement. Samples in solution were prepared by dissolving complexes in MeCN and the measurements were performed in J-1000 series JASCO CD spectrometer under room temperature.

Other Physical Measurements. Thermogravimetric analysis (TGA) was performed on a DTU-2A equipment at 300-750 K with a heating rate of 10 K min^{-1} in an air atmosphere. A Polycrystalline sample was used, and the sample degradation was observed after 550 K. Temperature-dependent UV-vis absorption spectra were obtained using a UV-3100 PC (shimadzu) scanning spectrophotometer with a helium-flow-type refrigerator. Powdered crystals were attached to the transparent tape. . DSC

measurements were performed on a Seiko EXSTAR 6000 instrument using cooling and heating rates of 10 K min^{-1}. Powder XRD analysis was performed on sample plates at room temperature at 50 kV and 300 mA using a Cu target (RIGAKU TTR-III).

A list of commercially available solvent and chemicals:

Product	Assay	Commercial source	
Acetone	99.5+%	FUJIFILM Wako Pure Chemical Co.	014-00347
Acetonitrile	99.5+%	FUJIFILM Wako Pure Chemical Co.	012-00387
Methanol	99.8+%	FUJIFILM Wako Pure Chemical Co.	139-01827
Ethanol	99.5+%	FUJIFILM Wako Pure Chemical Co.	055-00457
Diethyl ether	99.5+%	FUJIFILM Wako Pure Chemical Co.	051-01157
N,N-Dimethylformamide, Super Dehydrated	99.5+%	FUJIFILM Wako Pure Chemical Co.	045-32365
2-Propanol	99.7+%	FUJIFILM Wako Pure Chemical Co.	166-04831
Ethylenediamine	99+%	FUJIFILM Wako Pure Chemical Co.	053-00936
60% Perchloric acid		FUJIFILM Wako Pure Chemical Co.	169-00725
Sodium borohydride	95+%	Tokyo Chemical Industry Co.	S0480
Sodium hydroxide	97+%	FUJIFILM Wako Pure Chemical Co.	198-18863
Nickel(II) diacetate tetrahydrate	98+%	FUJIFILM Wako Pure Chemical Co.	146-00995
Sodium perchlorate	95+%	FUJIFILM Wako Pure Chemical Co.	192-09255
tetra-n-butylammonium bromide	98+%	FUJIFILM Wako Pure Chemical Co.	207-04335
Sodium d-tartrate dihydrate	99+%	FUJIFILM Wako Pure Chemical Co.	190-03455
Sodium oxalate	99.5+%	FUJIFILM Wako Pure Chemical Co.	198-02655
Sodium cyanide	97+%	FUJIFILM Wako Pure Chemical Co.	196-01855
Gallium (III) Chloride, Anhydrous	98+%	Tokyo Chemical Industry Co.	G0359
Cobalt(II) acetate tetrahydrate	99+%	FUJIFILM Wako Pure Chemical Co.	030-03602
Ammonium hexafluorophosphate	95-102%	FUJIFILM Wako Pure Chemical Co.	018-12491
3,5-dihydroxy-1,4-benzoquinone	98%	Sigma-Aldrich	195464
Triethylamine	99+%	FUJIFILM Wako Pure Chemical Co.	202-02646
Potassium Hexafluorophosphate	95.0-110.0 %	Tokyo Chemical Industry Co.	P1023
Silver Hexafluorophosphate	98+%	Tokyo Chemical Industry Co.	S0981

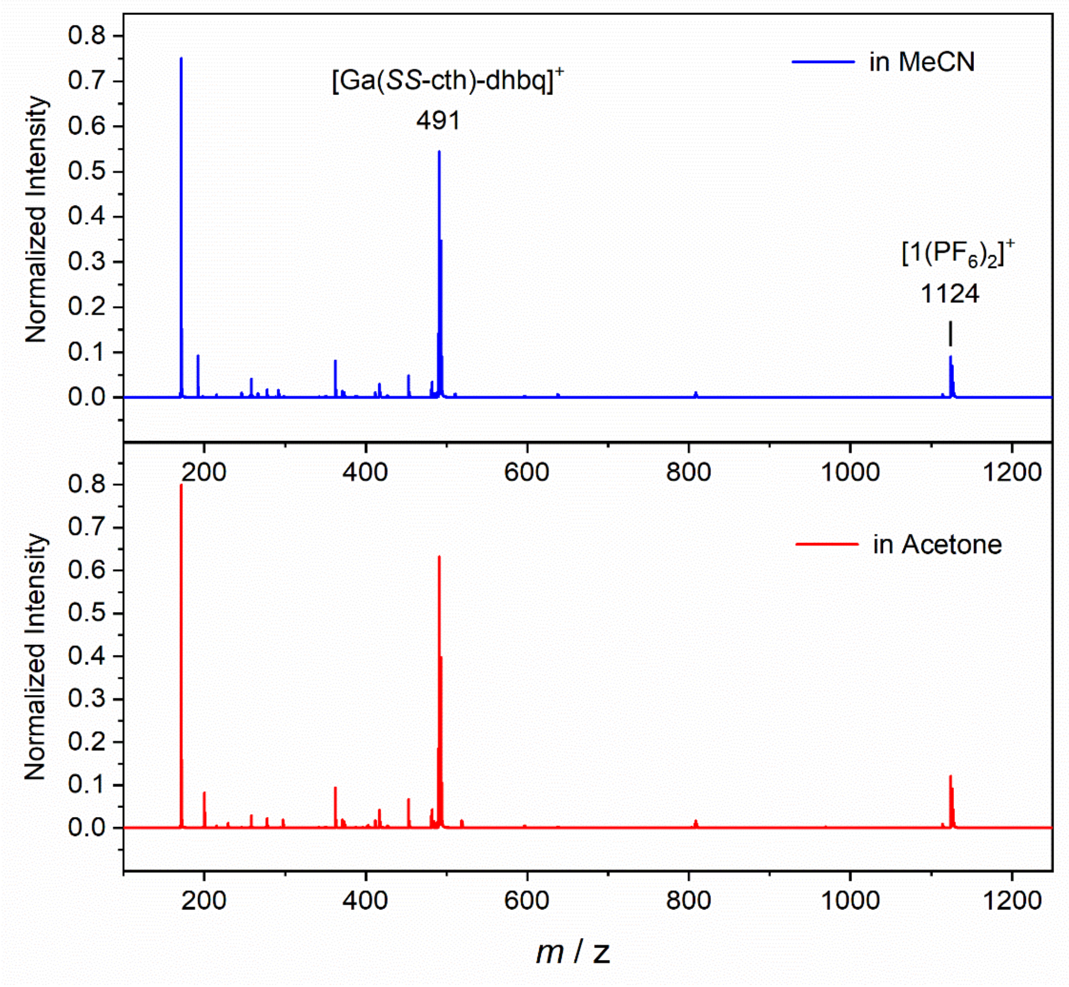

Supplementary Fig. 1. ESI-Mass spectroscopy data of the complex **1(PF$_6$)$_3$** in acetonitrile and acetone. Peak at *m/z* = 1124 indicates the molecular peak.

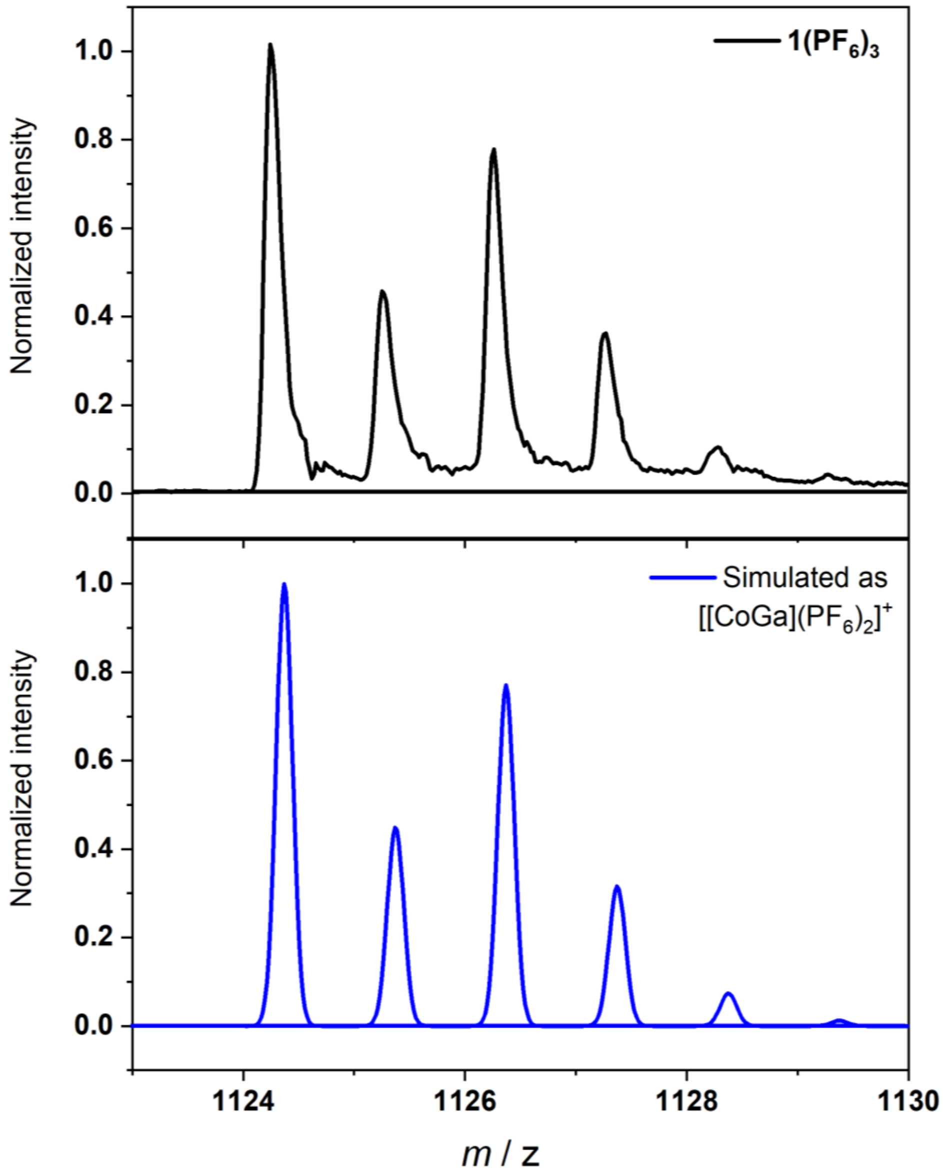

Supplementary Fig. 2. Isotopic distribution pattern for **$1(PF_6)_3$** (black) in acetone and calculated peaks for $[1(PF_6)_2]^+$ (blue). Absence of corresponding peaks for [CoCo] and [GaGa] in solution indicates selective crystallization of the [CoGa] species.

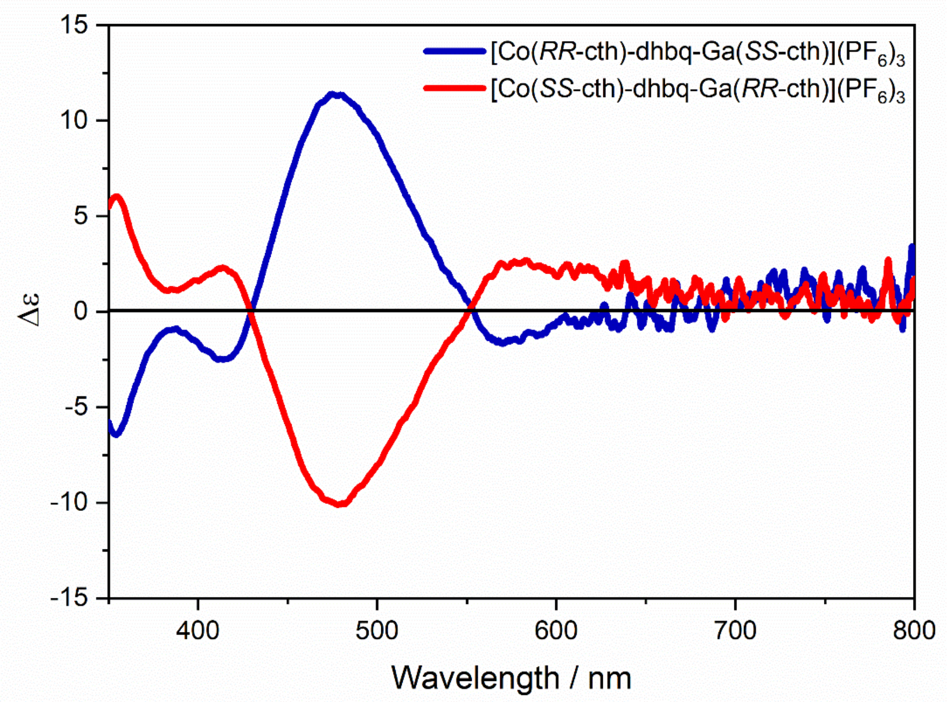

Supplementary Fig. 3. Circular dichroism spectra of **1(PF₆)₃** and its enantiomer showing mirror-image relationship in solution phase.

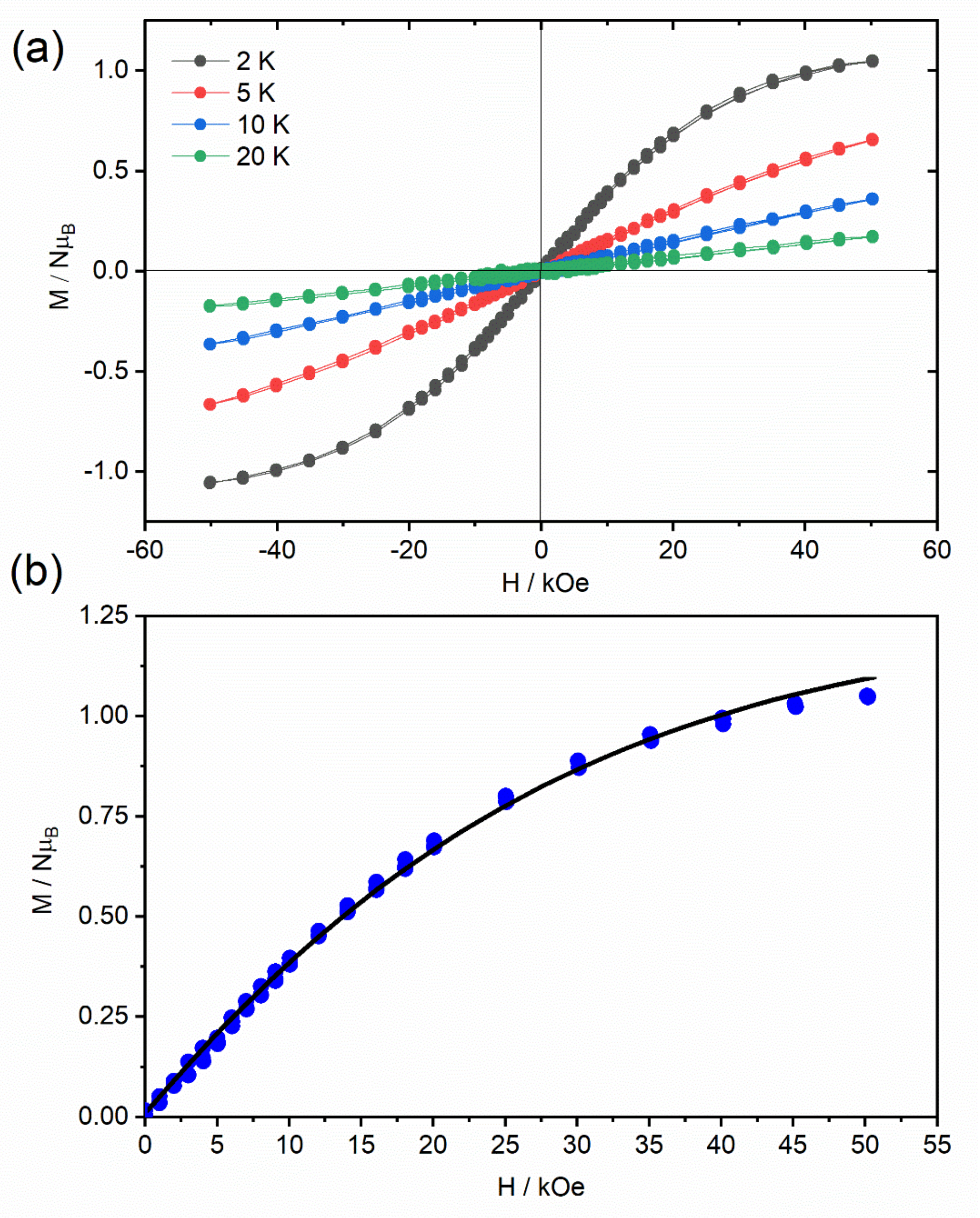

Supplementary Fig. 4. Magnetic field dependent magnetization of [CoGa] crystals. (a) *M* vs *H* plot measured at 2, 5, 10 and 20 K. (b) Magnetic field dependence at 2 K (blue dots) was fitted with Curie-Brillouin Law, $M = NgJ\mu_B B_J(x)$; where $x = gJ\mu_B B/k_B T$; *J* is the total angular momentum, *B* is Bohr magneton, *N* is Avogadro number, k_B is Boltzmann constant and *g* is the lande g-factor. The black line is the fitting line with the parameters *S* = ½, *g* = 2.0996 and *T* = 2 K.

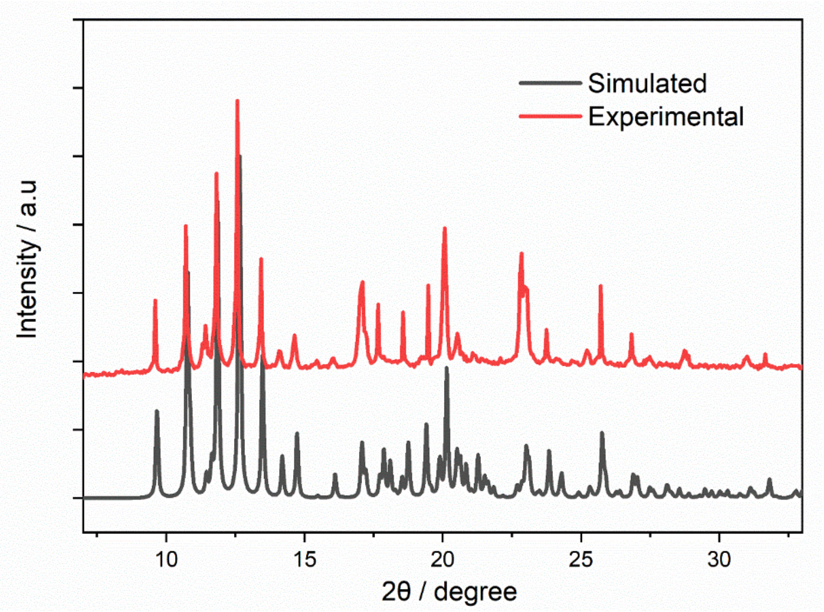

Supplementary Fig. 5. Powder X-ray diffraction pattern of [CoGa] at room temperature. Comparing experimental and simulated peaks indicates bulk phase purity of the polycrystalline sample.

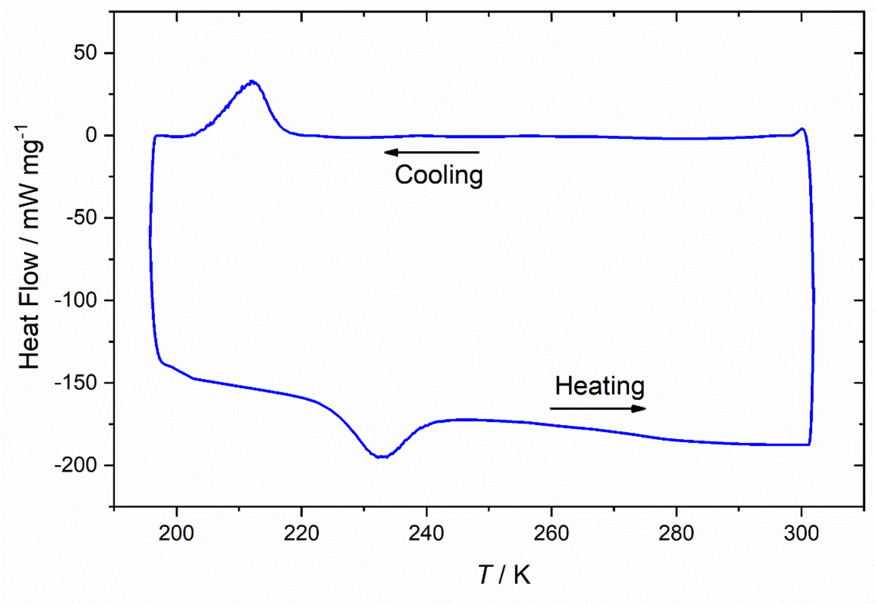

Supplementary Fig. 6. DSC curves of [CoGa] crystals in the heating and cooling cycle. Endothermic peak obtained at 233.1 K and exothermic peak at 212.2 K corresponds to the magnetic susceptibility measurement. The enthalpy difference (ΔH) was estimated to be 13 kJ mol^{-1} in the cooling run. The entropy change (ΔS) was calculated to be 64 J K^{-1} mol^{-1} using the equation $\Delta S = \Delta H/T_{1/2}$; where $T_{1/2}$ is the critical temperature of the phase transition (~213 K for cooling mode).

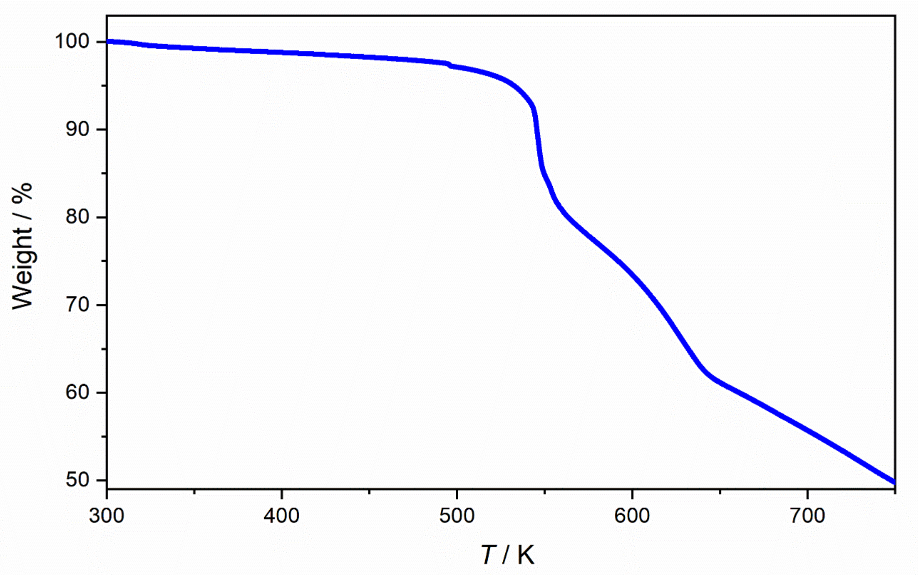

Supplementary Fig. 7. Thermogravimetric analysis of [CoGa] crystals. Thermal stability up to 530 K indicates there is no solvent molecule in the crystal lattice.

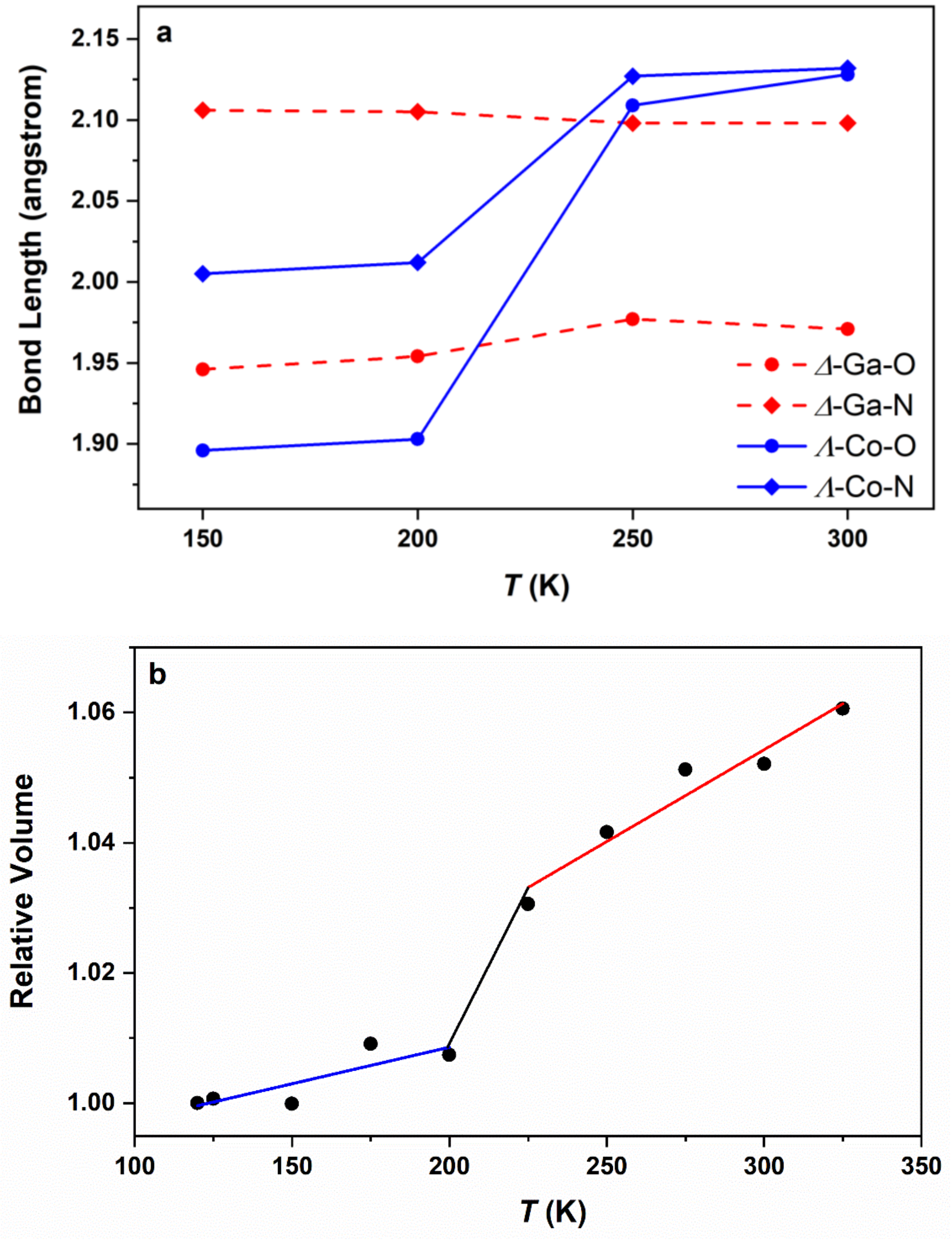

Supplementary Fig. 8. (a) Change in metal-ligand coordination bond-length of **1(PF_6)$_3$** with temperature evolution. (b) Change in relative volume of the complex with temperature.

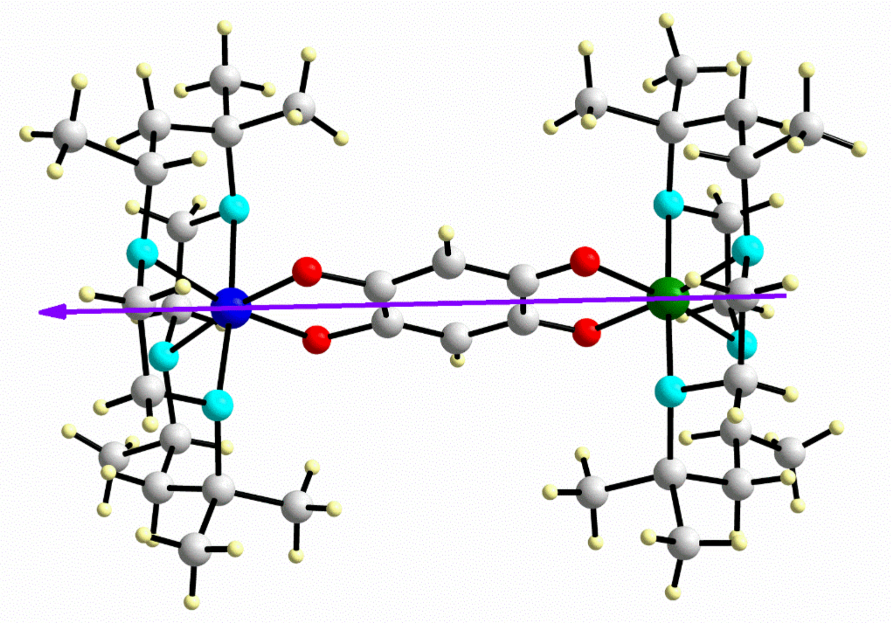

Supplementary Fig. 9. Molecular structure and calculated direction of the molecular dipole moment of **1(PF_6)$_3$** at the HS state. [Co (green), Ga (blue), O (red), C (gray), H (light yellow)].

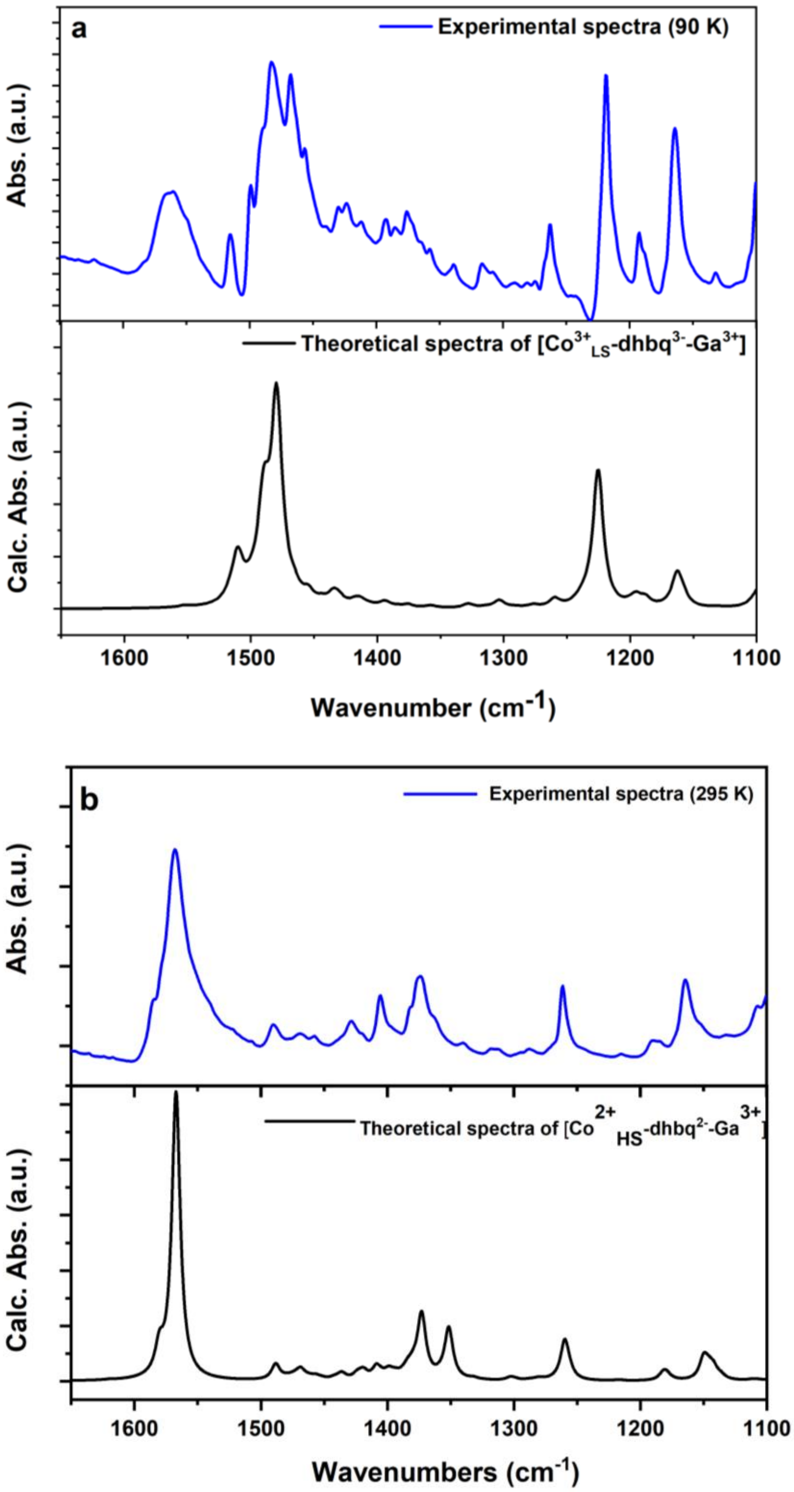

Supplementary Fig. 10. Comparison between experimentally and theoretically obtained infrared spectra. (a) Comparison of IR spectra at low temperature phase. (b) Comparison of IR spectra at high temperature phase.

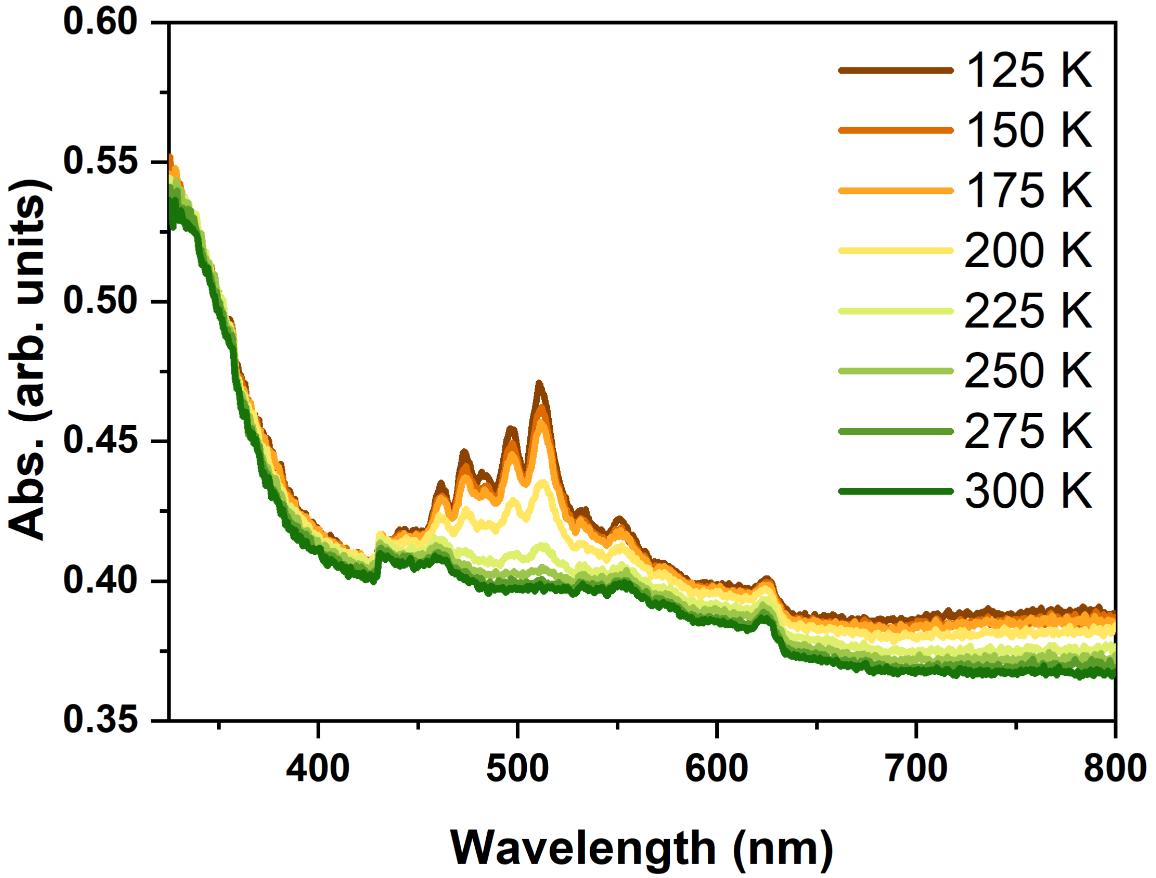

Supplementary Fig. 11. UV-Vis spectroscopic data for **1(PF_6)$_3$**. Temperature dependence of the absorption spectra in the visible region measured over a cooling course from 300 K to 125 K.

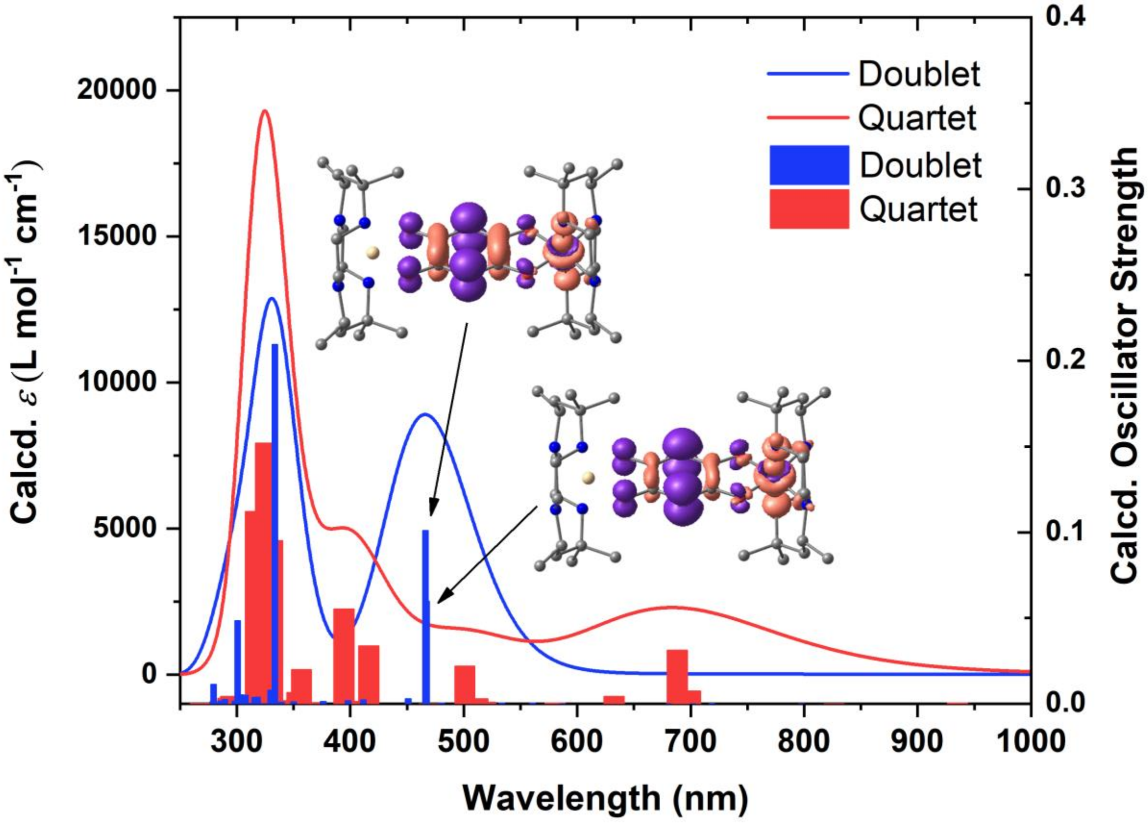

Supplementary Fig. 12. TD-DFT calculation results for [CoGa] compound. Red and blue lines correspond to quartet and doublet states, respectively (Y-axis on the left: Calcd. ε). Red and blue vertical bars correspond to oscillator strength of quartet and doublet states, respectively (Y-axis on the right: Calcd. Oscillator Strength).

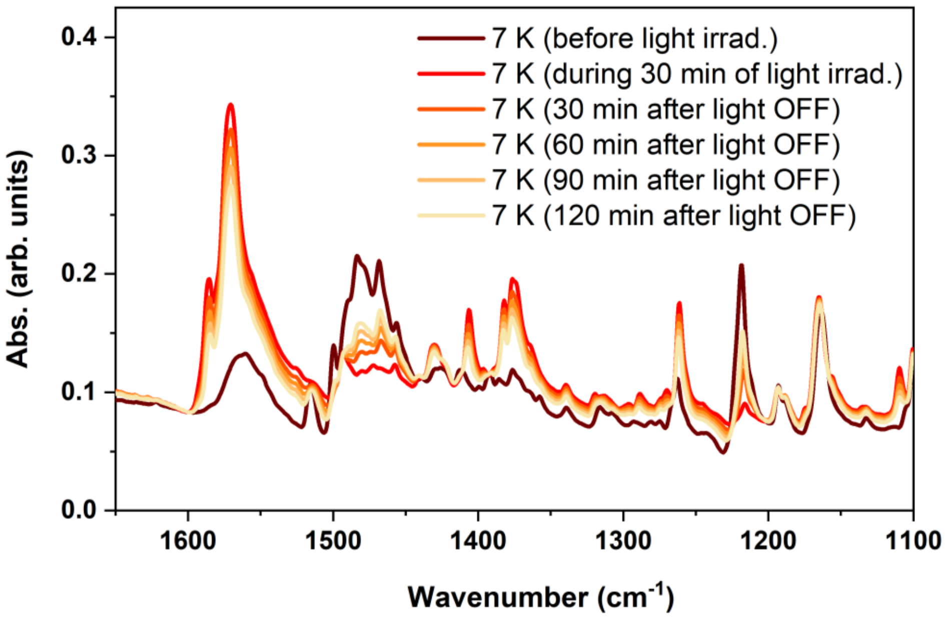

Supplementary Fig. 13. IR spectra of the [CoGa] complex at low temperature to demonstrate the presence of light-induced and long-lived metastable state.

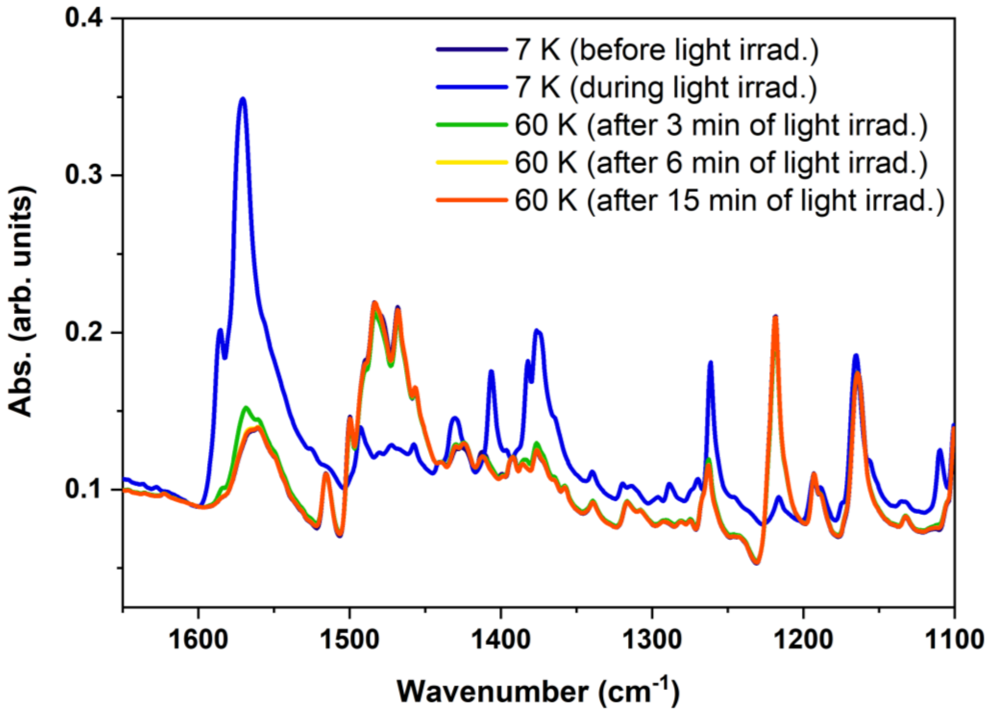

Supplementary Fig. 14. IR spectra of [CoGa] complex to demonstrate the quick relaxation of light-induced metastable state at elevated temperature.

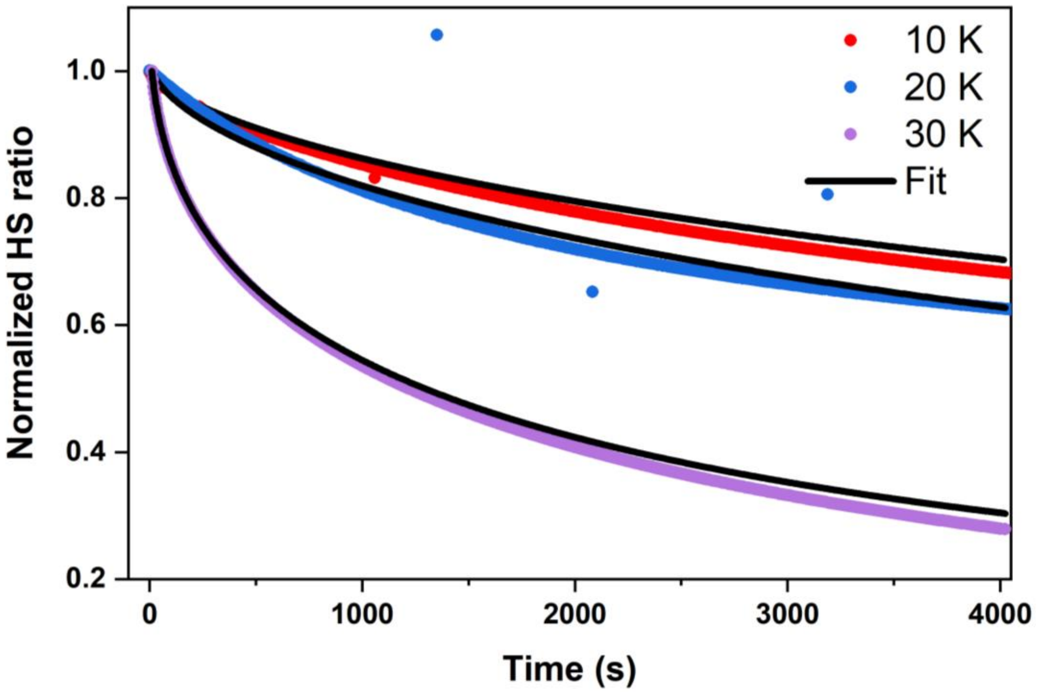

Supplementary Fig. 15. Relaxation from the light excited state to the ground state at 10, 20 and 30 K represented in terms of normalized high-spin ratio (γ_{HS}) *vs.* time (t) plot. Solid black line indicate the fitting by stretched exponential law: $\gamma_{HS}(t) = (1-\gamma_0)\exp[-(t/\tau_0)^{\beta}] + \gamma_0$, where γ_0 is the ratio of remnant high-spin species, τ_0 is the characteristics relaxation time and β is the distribution parameter. The fitting relaxation times are 35025(55) s with a distribution parameter of 0.49(1) for 10 K, 14112(32) s with a distribution parameter of 0.54(3) for 20 K and 2082(11) s with a distribution parameter of 0.47(2) for 30 K. We can conclude that the metastable state is very long-lived compared to similar dinuclear systems previously reported.

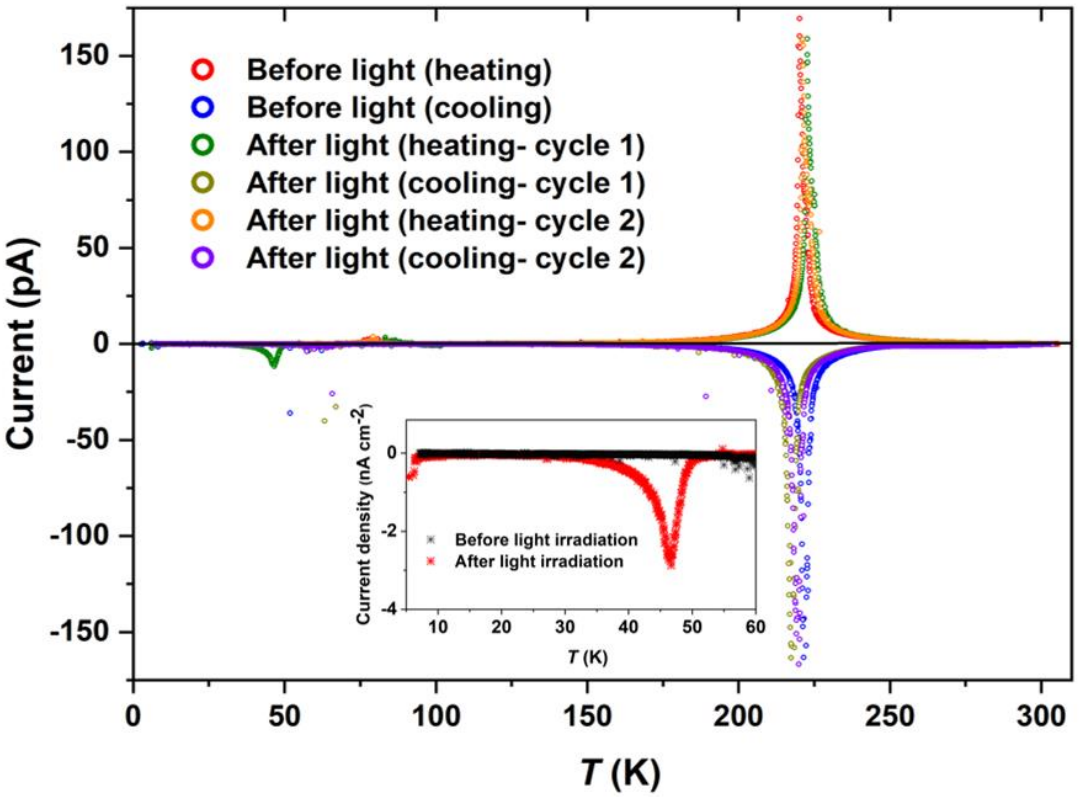

.

Supplementary Fig. 16. Measurements of pyroelectric current response before and after light irradiation in between 5 and 310 K. Inset- Current density vs. *T* plot in between 5 – 60 K after the light irradiation.

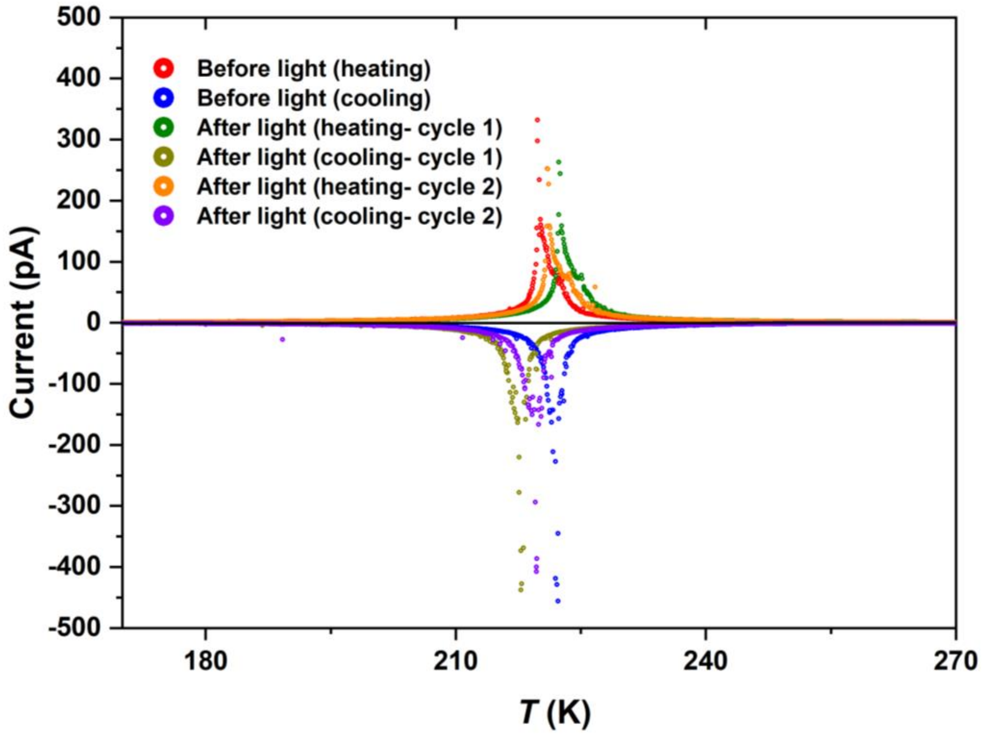

Supplementary Fig. 17. Current vs. Temperature plots around valence tautomeric transition temperature before and after light irradiation (heating - cooling - irradiation - heating 1 - cooling 1 - heating 2 - cooling 2).

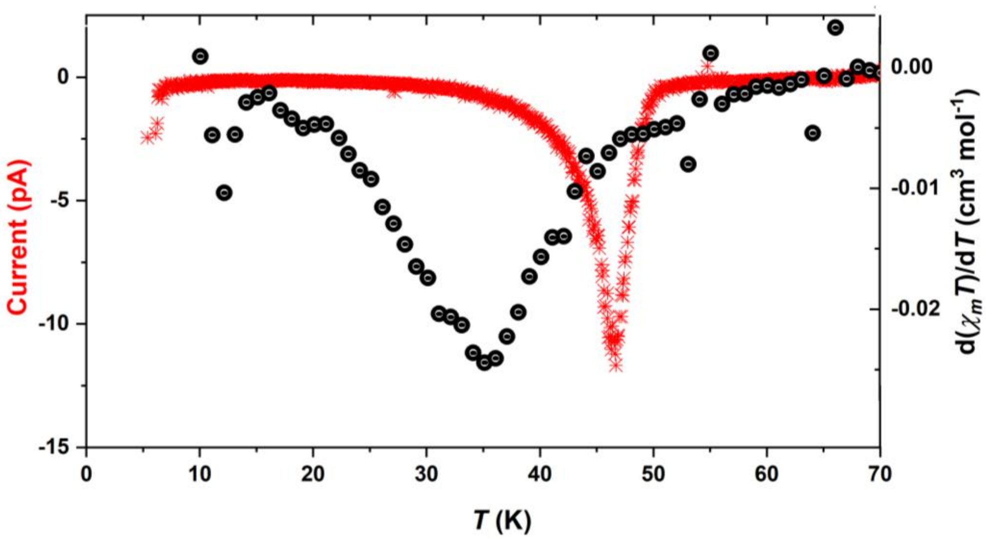

Supplementary Fig. 18. Correlation between magnetic properties and current release at low temperature (5-70 K) recorded after the light irradiation. The difference between the observed peaks is originating from the different experimental conditions of magnetic and pyroelectric measurements. The magnetic measurements were done on crystalline powder sample while during the pyroelectric measurements a well-shaped single-crystal was used. The scan rate of magnetic and pyroelectric measurements is 1 K min^{-1} and 5 K min^{-1}, respectively.

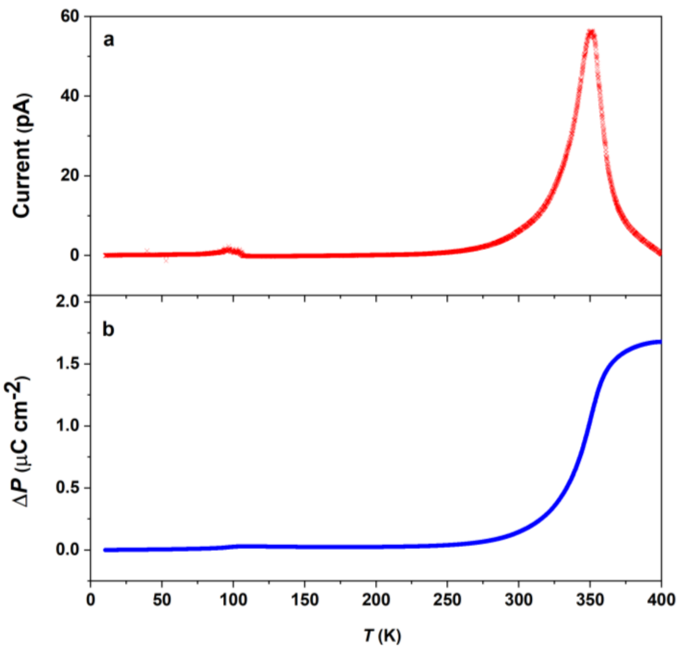

Supplementary Fig. 19. Measurement of pyroelectric properties of $[CrCo](PF_6)_3$ before light irradiation with temperature scan rate of 5 K min^{-1}. (a) Current vs. temperature plot recorded in between 10 K and 400 K. (b) Change in polarization with respect to temperature.

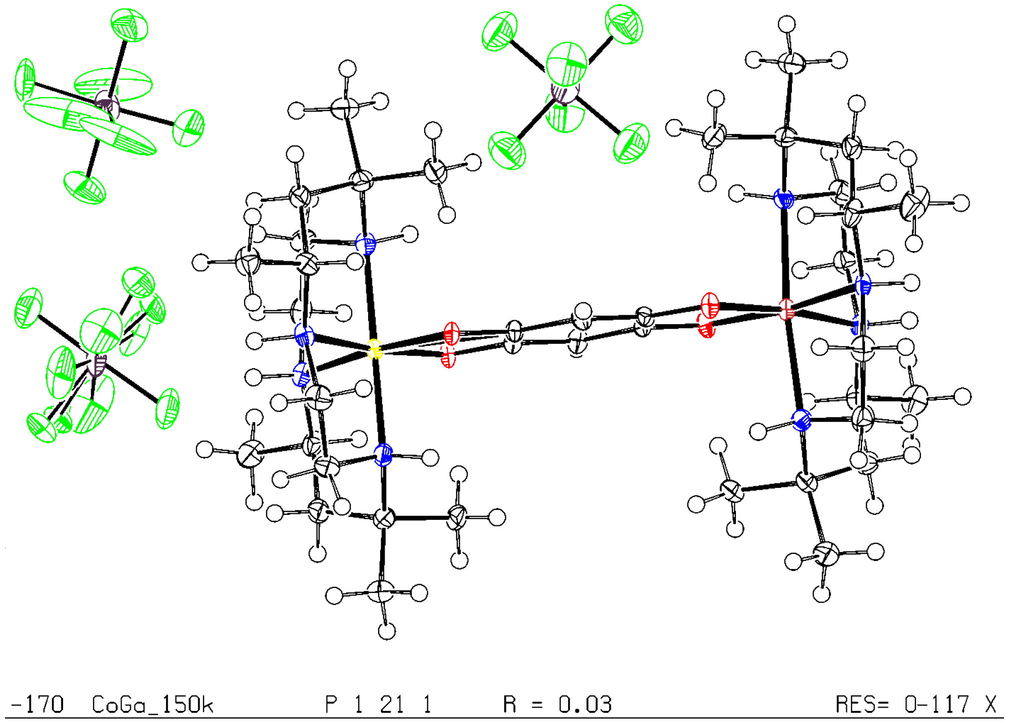

Supplementary Fig. 20. Structural figure of [CoGa] crystal with probability ellipsoids at 150 K.

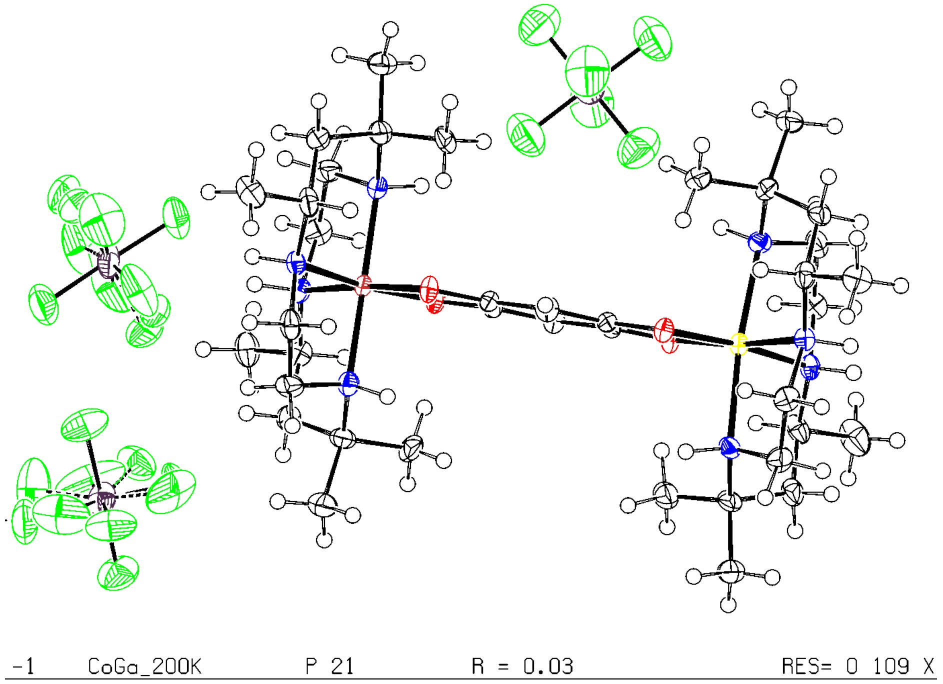

Supplementary Fig. 21. Structural figure of [CoGa] crystal with probability ellipsoids at 200 K.

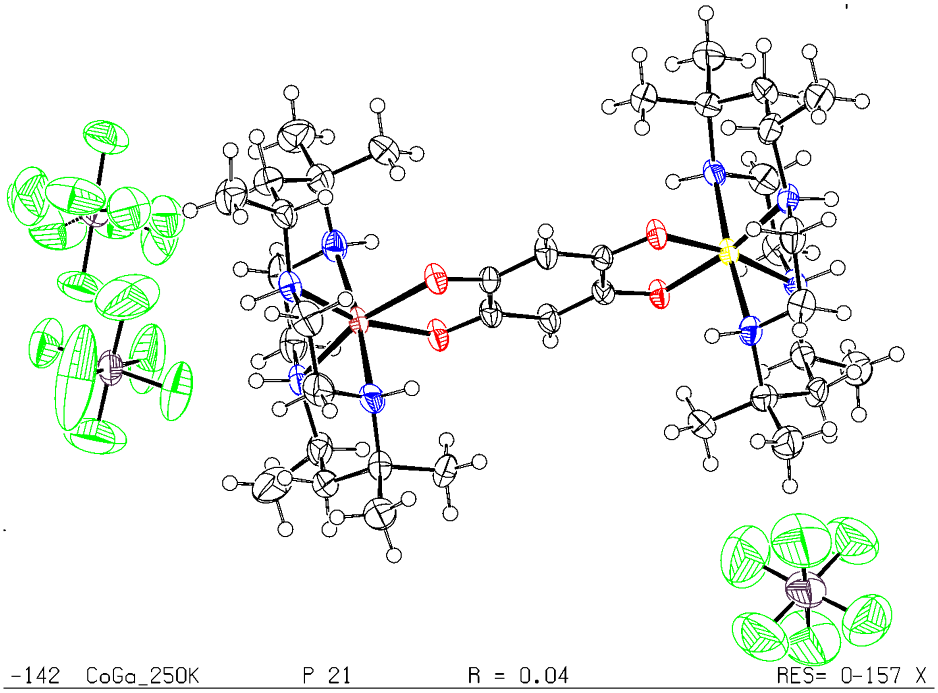

Supplementary Fig. 22. Structural figure of [CoGa] crystal with probability ellipsoids at 250 K.

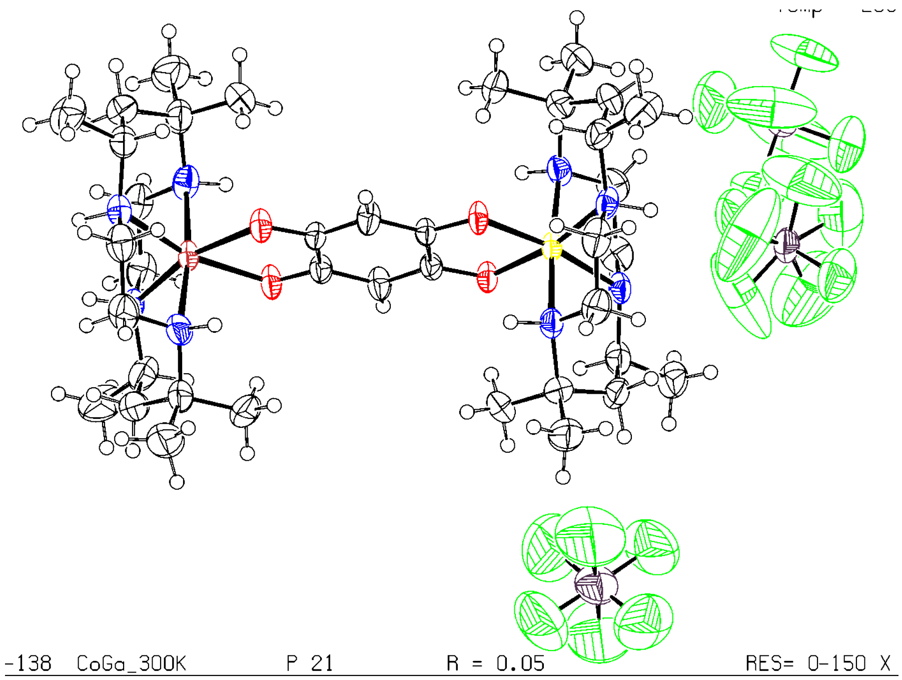

Supplementary Fig. 23. Structural figure of [CoGa] crystal with probability ellipsoids at 300 K.

Supplementary Table 1 | Crystallographic Data Collection and Structural Refinement Information for $1(PF_6)_3$

	$1(PF_6)_3$			
Empirical Formula	$C_{38}H_{74}CoF_{18}GaN_8O_4P_3$			
Formula Weight	1269.45			
Temperature(K)	150	200	250	300
Crystal System	*monoclinic*	*monoclinic*	*monoclinic*	*monoclinic*
Lattice Type	Primitive	Primitive	Primitive	Primitive
Space group	$P2_1$(#4)	$P2_1$(#4)	$P2_1$(#4)	$P2_1$(#4)
a(Å)	8.8848(2)	8.9077(3)	9.0509(3)	9.0940(3)
b(Å)	30.0850(6)	30.1398(9)	30.8198(6)	30.9284(6)
c(Å)	10.2129(3)	10.2433(3)	10.2009(3)	10.2054(3)
α(deg.)	90	90	90	90
β(deg.)	110.054(3)	110.035(4)	110.058(4)	110.118(4)
γ(deg.)	90	90	90	90
V (Å^3)	2564.39(12)	2583.66(15)	2671.30(14)	2695.27(15)
Z value	2	2	2	2
D_{calc}(g/cm^3)	1.667	1.634	1.622	1.608
radiation	MoKα (λ = 0.71073)			
R_1 (I>2.00s(I))	0.0343	0.0326	0.0390	0.0521
wR_2 (*all*)	0.0876	0.0796	0.1024	0.1471
GOF	1.021	1.019	1.016	1.046
Flack parameter	0.014(4)	0.019(5)	0.010(4)	0.021(4)
CCDC	2149695	2149698	2149697	214966

$^aR_1 = \Sigma||Fo| - |Fc||/\Sigma|Fo$. $^bR_2 = [\Sigma\{w(Fo^2 - Fc^2)^2\}/\Sigma\{w(Fo^2)^2\}]^{1/2}$

Supplementary Table 2 | Selected Bond Lengths (Å) for $1(PF_6)_3$

	$1(PF_6)_3$			
	150 K	200 K	250 K	300 K
Co1-O1 (Å)	1.898(5)	1.903(4)	2.109(4)	2.121(3)
Co1-O2 (Å)	1.891(5)	1.903(5)	2.111(4)	2.133(4)
Co1-N1 (Å)	1.990(4)	1.999(5)	2.115(5)	2.113(4)
Co1-N2 (Å)	2.018(5)	2.026(5)	2.155(5)	2.152(4)
Co1-N3 (Å)	1.999(5)	2.001(5)	2.105(5)	2.114(5)
Co1-N4 (Å)	2.016(5)	2.021(5)	2.141(5)	2.151(5)
Ga1-O3 (Å)	1.946(4)	1.951(4)	1.975(4)	1.979(4)
Ga1-O4 (Å)	1.950(5)	1.957(5)	1.974(4)	1.963(4)
Ga1-N5 (Å)	2.082(4)	2.078(5)	2.058(5)	2.061(4)
Ga1-N6 (Å)	2.125(5)	2.122(5)	2.126(5)	2.121(5)
Ga1-N7 (Å)	2.095(5)	2.095(5)	2.086(4)	2.084(5)
Ga1-N8 (Å)	2.119(4)	2.130(5)	2.111(5)	2.124(4)

Supplementary Table 3 | Control experiments for current generation.

**sample no.**	**system no.**	**compound name**	**space group**	**Polar/ Non-polar**	**presence of LIVT presence/ absence**	**current generation: yes/no**	**Ref.**
1	crystal 1, $\mathbf{1(PF_6)_3}$	$[CoGa](PF_6)_3$	$P2_1$	Polar	present	yes	
2	control 1	$[CoCo](PF_6)_3$	$P2_1/c$	Non-polar	present	no	Ref.[15,16]
3	control 2	$[CrCo](PF_6)_3$	$P2_1$	Polar	almost absent	no (below detection)	Ref.[16]

Supplementary Note 1.

The ligand field splitting and slater reduction obtained from the simulation of the Co pre-edge at 300 K are similar to characterizations obtained for other valence tautomeric Co systems. For instance L2,3-edge XAS measurements and ligand field multiplet simulations of $[(Tp)Fe(CN)_3Co-(PY5Me_2)]^+$ gave a 10Dq of 1.0 eV and slater reduction factor of 0.7.[17] M2,3-edge XAS measurements and ligand field multiplet simulations on the monomeric valence tautomer, $Co(Cat-N-BQ)_2$, gave a 10Dq of 1.5 eV and a slater reduction factor of 0.8.[18] Since these measurements were all conducted at different absorption edges they may exhibit different sensitivity.

Supplementary Note 2.

Pyroelectric coefficient (p) and polarization change (ΔP) was calculated with respect to the raw current data (Supplementary Fig. 16) according to the following equations-

Pyroelectric current, $i_p = pA(\delta T/\delta t)$, where A is the area of the measured surface

Pyroelectric coefficient, $p = (\delta P/\delta T)$, where P is denoted as macroscopic polarization

Calculation of polarization change during the electron transfer process-

T (K)	θ_1 (degree)	θ_2 (degree)	μ_{total} (debye)	V_{cell} (Å^3)	ΔP (μC cm^{-2})
150	21.44	21.44	0.21	2564.39	0
300	21.63	21.63	18.32	2695.27	2.05

1. θ_1, θ_2 are the angle between the dipole moments of the neighboring [CoGa]motif 1 and [CoGa]motif 2 and the crystalline *b*-axis calculated from single-crystal measurements (as per Supplementary Fig. 24).

2. μ_{total} is the sum of dipole moments of the unit cell (obtained from DFT calculations) projected on the crystalline *b*-axis; $\mu_{total} = \mu_{[CoGa]motif1} \cos\theta_1 + \mu_{[CoGa]motif2} \cos\theta_2$

3. V_{cell} is the volume of the unit cell at the given temperature.

4. ΔP is the polarization change; $\Delta P(T) = \mu_{total}(T)/V_{cell}(T) - \mu_{total}(150\ K)/V_{cell}(150\ K)$.

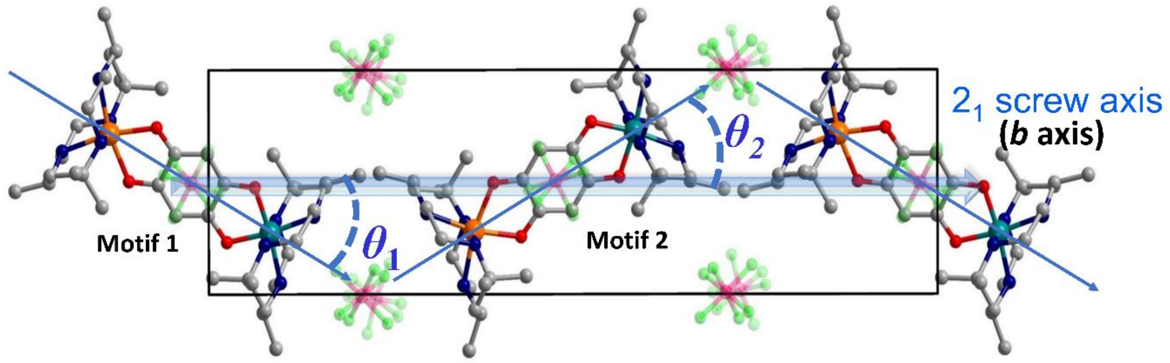

Supplementary Fig. 24. Crystal packing diagram of the [CoGa] complex from crystallographic *c*-axis. Blue arrows represent the direction of molecular dipole moments (color code: cobalt- orange, gallium- dark green).

Supplementary Note 3.

To compare the pyroelectric properties of our non-ferroelectric molecular material with previously reported state-of-the-art ferroelectrics, the most fundamental way is the comparison of Δ*P* (change in spontaneous polarization value). As it is considered, pyroelectric coefficient, $p = (\delta P/\delta T)$, where *P* is denoted as macroscopic polarization. Firstly, the property of thermally induced polarization switching in [CoGa] crystals is compared with other conventional molecular ferroelectrics.

Comparison with conventional molecular ferroelectrics-

Complex	Δ*P* (μC cm^{-2})	Ref.
Croconic acid	~21	19
P(VDF-TrFE)	~10	20
TGS	~3.8	21
**[CoGa]$(PF_6)_3$**	~2.9 (thermal stimuli)	**This article**

[CoGa]$(PF_6)_3$ exhibits large thermally induced polarization change compared with other non-ferroelectric type molecular pyroelectrics (where poling electric field is not required beforehand). The table is attached here for a comparison.

Comparison with currently emerging molecular non-ferroelectrics-

Complex	Δ*P* (μC cm^{-2})	Publishing year	Ref.
[Co(phendiox)(rac-cth)]$(ClO_4)\cdot 0.5EtOH$	~1.3	2020	22
[FeCo]$(PF_6)_3$	~1.1	2021	23
[CrCo]$(PF_6)_3$	~1.7	-	This article
**[CoGa]$(PF_6)_3$**	~2.9 (thermal stimuli)	-	**This article**

Supplementary Note 4.

The relaxation time of the photoinduced metastable state of the [CoGa] complex was compared to literature reports of closely related systems-

Complex	Relaxation time (s)	Distribution parameter	Ref.
[CoGa](PF_6)$_3$	35025 (10 K)<br>14112 (20 K)<br>2082 (30 K)	β = 0.49<br>β = 0.54<br>β = 0.47	This article
[CoCo](PF_6)$_3$	13100 (10 K)<br>5900 (20 K)<br>2450 (30 K)	β ~ 0.5 to 0.7	24
[Co(phen)(3,5-dBSQ)]	5760 (5 K)<br>2820 (15 K)<br>660 (35 K)	-	25
[{Co(dpqa)}$_2$(dhbq)](PF_6)$_3$	7692 (20 K)	β = 0.6	26
[Co(phendiox)(rac-cth)](ClO_4)·0.5EtOH	1158 (7 K)<br>392 (20 K)	β = 0.74<br>β = 0.57	22

After comparing with these mononuclear and dinuclear LIVT systems, it is evident that the lifetime of the metastable state is high enough in the case of [CoGa] system which is a favorable factor for energy conversion prospect.

Supplementary Note 5.

CheckCIF alerts associated with CCDC 2149695, 2149698 and 214966-

Alert B - PLAT987 ALERT 1 B The Flack x is >> 0 - Do a BASF/TWIN Refinement Please Check

Although the Flack parameter close to zero indicates the material is enantiopure and the atomic coordinates determined from the data correspond to the actual structure, slight deviations might arise from defects in the crystal. On a further note, Flack and Bernardinelli concluded that for a material known to be enantiopure, a standard uncertainty of less than 0.08 is sufficient for the

assignment of absolute structure to be well determined.[27] (Ref. Flack, H.; Bernardinelli, G. Reporting and evaluating absolute-structure and absolute-configuration determinations. *J. Appl. Cryst.* 2000, 33, 1143–1148.)

Flack parameters of 0.010 - 0.021 of these crystal data are low enough and very common in similar molecular crystals. Therefore, no additional treatments are required.

References:

1 Sheldrick, G. M. Crystal structure refinement withSHELXL. *Acta Crystallographica Section C Structural Chemistry* **71**, 3-8 (2015).

2 Dolomanov, O. V. OLEX2: a complete structure solution, refinementand analysis program. *Journal ofAppliedCrystallography* **42**, 339-341 (2009).

3 Retegan, M. Crispy: c0.7.3, 2019.

4 Haverkort, M. W., Zwierzycki, M. & Andersen, O. K. Multiplet ligand-field theory using Wannier orbitals. *Physical Review B* **85**, 165113 (2012).

5 Gaussian 09, Revision E.01, Frisch, M. J.; Trucks, G. W.; Schlegel, H. B.; Scuseria, G. E.; Robb, M. A.; Cheeseman, J. R.; Scalmani, G.; Barone, V.; Mennucci, B.; Petersson, G. A.; Nakatsuji, H.; Caricato, M.; Li, X.; Hratchian, H. P.; Izmaylov, A. F.; Bloino, J.; Zheng, G.; Sonnenberg, J. L.; Hada, M.; Ehara, M.; Toyota, K.; Fukuda, R.; Hasegawa, J.; Ishida, M.; Nakajima, T.; Honda, Y.; Kitao, O.; Nakai, H.; Vreven, T.; Montgomery, J. A., Jr.; Peralta, J. E.; Ogliaro, F.; Bearpark, M.; Heyd, J. J.; Brothers, E.; Kudin, K. N.; Staroverov, V. N.; Kobayashi, R.; Normand, J.; Raghavachari, K.; Rendell, A.; Burant, J. C.; Iyengar, S. S.; Tomasi, J.; Cossi, M.; Rega, N.; Millam, J. M.; Klene, M.; Knox, J. E.; Cross, J. B.; Bakken, V.; Adamo, C.; Jaramillo, J.; Gomperts, R.; Stratmann, R. E.; Yazyev, O.; Austin, A. J.; Cammi, R.; Pomelli, C.; Ochterski, J. W.; Martin, R. L.; Morokuma, K.; Zakrzewski, V. G.; Voth, G. A.; Salvador, P.; Dannenberg, J. J.; Dapprich, S.; Daniels, A. D.; Farkas, Ö.; Foresman, J. B.; Ortiz, J. V.; Cioslowski, J.; Fox, D. J. Gaussian, Inc., Wallingford CT, 2009.

6 Wachters, A. J. H. Gaussian Basis Set for Molecular Wavefunctions Containing Third-Row Atoms. *The Journal of Chemical Physics* **52**, 1033-1036 (1970).

7 Hay, P. J. Gaussian basis sets for molecular calculations. The representation of 3d orbitals in transition-metal atoms. *The Journal of Chemical Physics* **66**, 4377-4384 (1977).

8 Krishnan, R., Binkley, J. S., Seeger, R. & Pople, J. A. Self-consistent molecular orbital methods. XX. A basis set for correlated wave functions. *The Journal of Chemical Physics* **72**, 650-654 (1980).

9 Dunning, T. H. J. & Hay, P. J. *Modern Theoretical Chemistry, Ed. Schaefer, H. F. III, Vol. 3* (Plenum, New York, 1977*)* 1-28.

10 Becke, A. D. Density-functional thermochemistry. III. The role of exact exchange. *The Journal of Chemical Physics* **98**, 5648-5652 (1993).

11 Reiher, M. Theoretical Study of the $Fe(phen)_2(NCS)_2$ Spin-Crossover Complex with Reparametrized Density Functionals. *Inorganic Chemistry* **41**, 6928-6935 (2002).

12 Bauernschmitt, R. & Ahlrichs, R. Treatment of electronic excitations within the adiabatic approximation of time dependent density functional theory. *Chemical Physics Letters* **256**, 454-464 (1996).

13 Casida, M. E., Jamorski, C., Casida, K. C. & Salahub, D. R. Molecular excitation energies to high-lying bound states from time-dependent density-functional response theory: Characterization and correction of the time-dependent local density approximation ionization threshold. *The Journal of Chemical Physics* **108**, 4439-4449 (1998).

14 Lu, T. & Chen, F. Multiwfn: A multifunctional wavefunction analyzer. *Journal of Computational Chemistry* **33**, 580-592 (2012).

15 Carbonera, C., Dei, A., Létard, J.-F., Sangregorio, C. & Sorace, L. Thermally and Light-Induced Valence Tautomeric Transition in a Dinuclear Cobalt–Tetraoxolene Complex. *Angewandte Chemie International Edition* **43**, 3136-3138 (2004).

16 Kanegawa, S. *et al.* Directional Electron Transfer in Crystals of [CrCo] Dinuclear Complexes Achieved by Chirality-Assisted Preparative Method. *Journal of the American Chemical Society* **138**, 14170-14173 (2016).

17 Sainctavit, P. *et al.* Atomic Scale Evidence of the Switching Mechanism in a Photomagnetic CoFe Dinuclear Prussian Blue Analogue. *J. Am. Chem. Soc.* **141**, 8, 3470–3479 (2019).

18 Vura-weis, J. *et al.* Photoinduced valence tautomerism of a cobalt-dioxolene complex revealed with femtosecond M-edge XANES. *J. Chem. Phys.* **151**, 104201 (2019).

19 Horiuchi, S. *et al.* Above-room-temperature ferroelectricity in a single-component molecular crystal. *Nature* **463**, 789-792, doi:10.1038/nature08731 (2010).

20 Tajitsu, Y., Ogura, H., Chiba, A. & Furukawa, T. Investigation of Switching Characteristics of Vinylidene Fluoride/Trifluoroethylene Copolymers in Relation to Their Structures. *Japanese Journal of Applied Physics* **26**, 554-560, doi:10.1143/jjap.26.554 (1987).

21 Matthias, B. T., Miller, C. E. & Remeika, J. P. Ferroelectricity of Glycine Sulfate. *Physical Review* **104**, 849-850, doi:10.1103/PhysRev.104.849.2 (1956).

22. Wu, S.-Q. et al. Macroscopic Polarization Change via Electron Transfer in a Valence Tautomeric Cobalt Complex. *Nature Communications* 11, 1992 (2020).

23. Sadhukhan, P. et al. Manipulating electron redistribution to achieve electronic pyroelectricity in molecular [FeCo] crystals. *Nature Communications* 12, 4836 (2021).

24. C. Carbonera *et al.* Relaxation dynamics of a photoinduced di-cobalt-tetraoxolene valence tautomer*Inorg. Chim. Acta*. **360**, 3825-3828 (2007).

25. O. Sato, S. Hayami, Y. Einaga, Z.-Z. Gu. Control of the Magnetic and Optical Properties in Molecular Compounds by Electrochemical, Photochemical and Chemical Methods *Bull. Chem. Soc. Jpn.* **76**, 443 (2003).

26. Y. Teki *et al.* ESR study of light-induced valence tautomerism of a dinuclear Co complex. *Eur. J. Inorg. Chem.* 3761-3767 (2011).

27. Flack, H.; Bernardinelli, G. Reporting and evaluating absolute-structure and absolute-configuration determinations. *J. Appl. Cryst.* 2000, 33, 1143–1148.)
